# INtraVESical immunoTherapy (INVEST) prior to radical cystectomy for bladder cancer: trial protocol

**DOI:** 10.1111/bju.70387

**Published:** 2026-07-27

**Authors:** Ruby Lister‐Whelan, Ethan Senior, Jessica Kendall, Jamie B. Oughton, Fiona Collinson, Jon Griffin, Rachel Hubbard, Steven Kennish, Aidan Noon, Derek J. Rosario, Irbaz B. Riaz, Julie Telliez, James W. F. Catto, Syed A. Hussain

**Affiliations:** ^1^ Clinical Trials Research Unit, Leeds Institute of Clinical Trials Research University of Leeds Leeds UK; ^2^ Division of Clinical Medicine, School of Medicine & Population Health University of Sheffield Sheffield UK; ^3^ Department of Histopathology Sheffield Teaching Hospitals NHS Foundation Trust Sheffield UK; ^4^ Department of Radiology Sheffield Teaching Hospitals NHS Foundation Trust Sheffield UK; ^5^ Department of Urology Sheffield Teaching Hospitals NHS Foundation Trust Sheffield UK; ^6^ Department of Oncology, Weston Park Cancer and Centre Sheffield Teaching Hospitals NHS Foundation Trust Sheffield UK; ^7^ Department of Hematology/Oncology Mayo Clinic Phoenix AZ USA; ^8^ F. Hoffmann‐La Roche Ltd. Basel Switzerland

**Keywords:** Atezolizumab, Tecentriq, radical cystectomy, bladder cancer, intravesical immunotherapy, Phase I, protocol, dose escalation, window of opportunity

## Abstract

**Background:**

High‐risk non‐muscle‐invasive bladder cancer (HRNMIBC) has a variable prognosis, managed predominantly by local surgical resection and intravesical Bacillus Calmette–Guérin (BCG), or radical cystectomy (RC). Current treatments are poorly tolerated, have supply limitations and often fail to control the disease. Treatments overcoming these limitations are needed to improve HRNMIBC outcomes. Many novel agents have been approved in recent years (including systemic pembrolizumab, nogapendekin alfa inbakicept‐pmln, N‐803), but long‐term efficacy is unknown. Given advanced bladder cancer findings, we believe direct intravesical administration of atezolizumab may aid with managing HRNMIBC or muscle‐invasive bladder cancer in patients who are ineligible for cisplatin‐based neoadjuvant chemotherapy.

**Study design:**

The INtraVESical immunoTherapy (INVEST) trial is a UK‐based, open‐label, non‐randomised, single‐centre, Phase Ib dose escalation, window of opportunity study exploring atezolizumab administration via intravesical routes (passive instillation and direct injection [sub‐urothelial]) in patients with bladder cancer with all stages undergoing RC. For both routes, an initial dose confirmation stage will determine toxicity and recommended atezolizumab dosage, followed by a dose expansion stage to further evaluate safety and toxicity at the recommended dose.

**Endpoints:**

We aim to investigate safety (primary) and preliminary activity (secondary) of intravesical atezolizumab delivered by passive instillation and direct injection into the tumour/bladder wall in patients with bladder urothelial cell carcinoma prior to RC. Primary endpoints include dose limiting toxicities and adverse events. Secondary endpoints include progression‐free survival, pathological complete response rate and treatment compliance. Exploratory endpoints include pharmacokinetic profile (assessed by serum and urine concentrations at prespecified timepoints), spatial analysis of atezolizumab distribution within RC tissue by imaging mass spectrometry and overall survival.

**Patients and Methods:**

Atezolizumab will be administered intravesically by either passive instillation or direct injection into the tumour/bladder wall. Cohorts will receive either 600 mg or 1200 mg doses administered as a single dose or as multiple doses (weekly for 3–6 weeks).

**Trial Registration:**

International Standard Randomised Controlled Trial Number: ISRCTN15842444, registered 2 December 2022.

**Abbreviations**
ADA: anti‐drug antibody; (S)AE, (serious) adverse event
AEoSI: AEs of special interest
(S)AR: (serious) adverse reaction
BCa: bladder cancer
CTCAE: Common Terminology Criteria for Adverse Events
CTLA‐4: cytotoxic T‐lymphocyte associated protein 4
CTRU: Clinical Trials Research Unit; DLT, dose‐limiting toxicity; (cf)(ct)DNA; (cell free) (circulating tumour) DNA
FDG: fluorodeoxyglucose
FFPE: formalin‐fixed paraffin‐embedded
ICI: immune checkpoint inhibitor
INVEST: INtraVESical immunoTherapy (trial)
ISRCTN: International Standard Randomised Controlled Trial Number
MIBC: muscle‐invasive bladder cancer
NCI: National Cancer Institute
(HR)NMIBC: (high‐risk) non‐muscle‐invasive bladder cancer
OS: overall survival
PBMCs: peripheral blood mononuclear cells
pCR: pathological complete response
PD‐1: programmed death 1
PD‐L1: programmed death‐ligand 1
PET: positron emission tomography
PFS: progression‐free survival
PK: pharmacokinetic
PPIE: patient and public involvement and engagement
RC: radical cystectomy
RD: recommended dose
SRC: Safety Review Committee
SUSAR: suspected unexpected serious AR
TMG: Trial Management Group
TURBT: transurethral resection of bladder tumour
UCC: urothelial cell carcinoma

## Background

Bladder cancer (BCa) is a common malignancy and one of the most expensive to treat [1, 2]. Most BCas are urothelial cell carcinoma (UCC) and stratified into non‐muscle‐invasive (NMIBC) and muscle‐invasive (MIBC) stages. NMIBC may be low or high risk (HRNMIBC) according to histological features. HRNMIBC is managed using local surgical resection and intravesical immunotherapy (e.g., BCG) with surveillance, or radical cystectomy (RC) [3, 4]. Most patients with HRNMIBC develop multiple relapses (local recurrence in up to 75%) and up to 25% develop progression to muscle invasion, metastases or death [5, 6]. Current treatments are laborious, poorly tolerated, have supply limitations, and fail to control the disease in many patients [7]. Novel treatments overcoming these limitations are urgently needed.

Numerous agents have been evaluated for HRNMIBC (including systemic pembrolizumab for BCG‐unresponsive cancers, subcutaneous sasanlimab for BCG‐naïve cancers, and intravesical TAR‐200, cretostimogene grenadenorepvec, nogapendekin alfa inbakicept‐pmln, nadofaragene firadenovec and N‐803 for both BCG‐naïve or unresponsive cancer) [8]. Some of the most promising include immune checkpoint inhibitors (ICIs) that target the programmed death‐ligand 1 (PD‐L1) axis, including systemic atezolizumab (known to be effective in advanced MIBC [9, 10]). Contemporary experience with systemic intravenous atezolizumab in metastatic disease shows good efficacy and few Grade 3–4 treatment‐related adverse reactions (ARs), with 7% of patients having experienced an adverse event (AE) leading to atezolizumab cessation [11, 12]. Given this toxicity profile and the natural history of HRNMIBC [4, 5], the authors have explored the potential of intravesical ICIs for HRNMIBC [13, 14, 15, 16]. The SUB‐urothelial DUrvalumab injEction‐1 (SUBDUE‐1) study recruited nine participants for direct intra‐tumoral injection of durvalumab (25, 75 and 150 mg) [14]. In all, 14 AEs were reported by six (67%) participants (including one Grade 3 AE). Transient elevation of perioperative thyroid‐stimulating hormone was seen in two participants. There were no ARs, and all participants underwent planned RC. Visible tumour was present in four participants, and one achieved pathological complete response (pCR). An increase in monocytes was seen at 150 mg dose. In a 3 + 3 Phase I trial of intravesical pembrolizumab and BCG in BCG‐unresponsive NMIBC (NCT02808143) [16] the 6‐month and 1‐year recurrence‐free rates were 67% and 22%, respectively, in nine participants with a median follow‐up of 35 months. The maximum tolerated dose was not reached; one dose‐limiting toxicity (DLT) was reported (Grade 2 diarrhoea, 22‐day duration) in the participant with the longest time to recurrence. One death occurred due to myasthenia gravis that was deemed potentially related to treatment.

These human trials are supported by translational science. Van Hooren et al. [17] found local delivery of anti‐cytotoxic T‐lymphocyte associated protein 4 (CTLA‐4) antibody was of similar efficacy to systemic administration, but with lower toxicity (measured by systemic CTLA‐4 antibody counts and interleukin 6 expression). Tumour regression could be enhanced with local anti‐programmed death 1 (PD‐1) antibody injection. Various other observations suggest locally administered (intravesical) atezolizumab may reach the site of T‐cell interactions with tumour neoantigens. For example, T cells are found in the superficial bladder wall (such as lamina propria) in direct contact with the urine, T cells are present in the urine of patients with BCa and change in response to immunotherapy [18], T‐cell populations can be primed to improve the efficacy of BCG, suggesting the need for tumour, BCG, and T‐cell interaction [19], tumours disrupt the bladder urine/barrier to allow escape and/or absorption of larger molecules (such as therapeutic antibodies); and agents such as BCG are endocytosed to reach deeper bladder tissues/regional nodes [19, 20].

Given that intravesical administration is a novel route, the safest, most tolerable, and most active dose for each agent is unknown. Within this trial, we will explore 600 and 1200 mg atezolizumab, reflecting prior researchers’ proposals, the volumes of agents needed to fill the bladder, a pragmatic choice around drug delivery and considering that the bladder will be removed shortly. Both routes of administration will start with the lower dose (600 mg), and for the direct injection route we will record how much of the agent can be injected into the tumour/adjacent bladder wall.

The majority of patients in clinical practice received 1200 mg intravenous atezolizumab administered every 3 weeks. In this trial we will use either 600 or 1200 mg up to once per week for 6 weeks in the multiple dose stage. We anticipate significantly less systemic absorption via passive bladder instillation and intratumoral routes of administration when compared with intravenous administration of atezolizumab, and so we expect fewer side effects and fewer problems with accumulation of drug effects. This is supported by data from the PemBla study (ClinicalTrials.gov identifier: NCT03167151) that evaluated 6‐weekly intravesical pembrolizumab instillations [21]. Six patients were treated with no DLTs and ARs were low grade. Pharmacokinetic (PK) and pharmacodynamic assays did not detect systemic pembrolizumab and no changes in peripheral immune cell populations were observed. Analysis of the immune infiltrate present in the tumour at the time of transurethral resection of bladder tumour (TURBT) prior to treatment confirmed higher PD‐1 expression on tumour infiltrating lymphocytes relative to those in the periphery, suggesting a degree of pre‐existing immune cell exhaustion and supporting targeting PD‐1/PD‐L1 interactions to augment anti‐tumour responses.

We hypothesise intravesical atezolizumab may be an effective, tolerable treatment for patients with bladder UCC undergoing RC. To understand the safety and tolerability of atezolizumab delivered intravesically, we are undertaking this window of opportunity trial prior to RC.

## Study Design

**Fig. 1:**
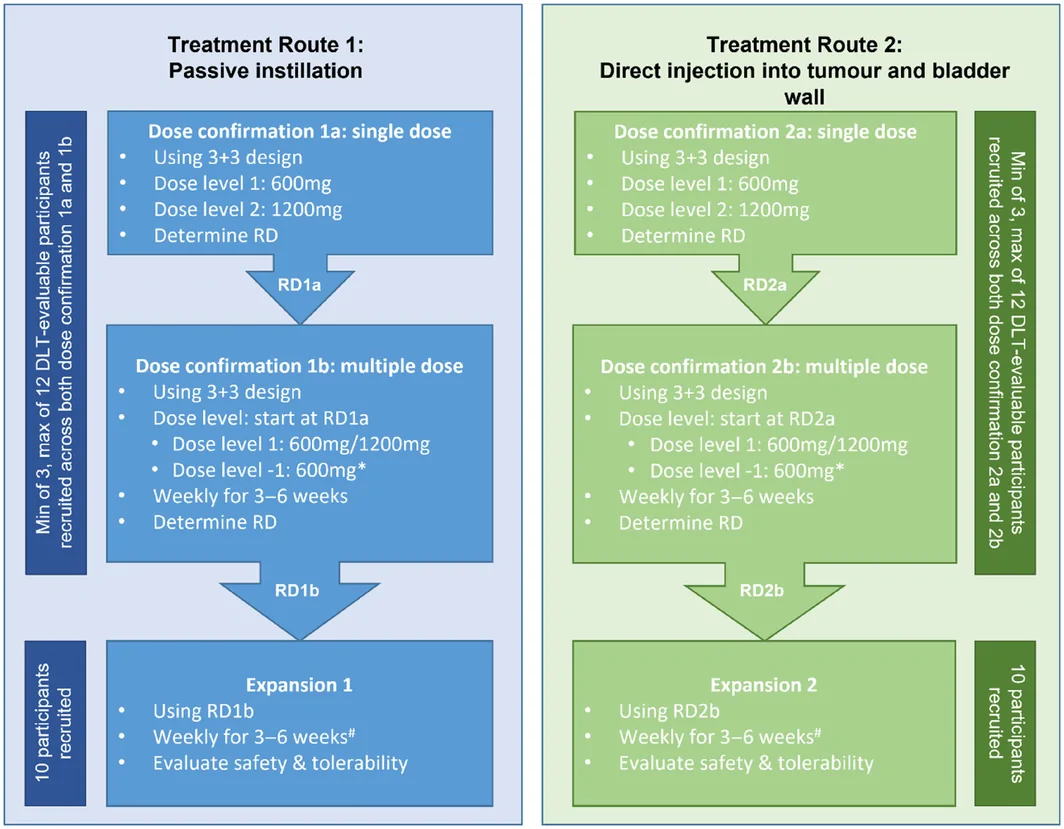
Trial flow diagram. The first single dose and multiple dose participants in each treatment route will be recruited and observed for the DLT reporting period (from first dose of trial treatment until RC), prior to any further participants being recruited to that respective first cohort. * Only if RD1a/RD2a is 1200 mg. # Single dose (RD1a/RD2a) if multiple doses are not tolerated in dose confirmation 1b/2b, respectively.

The INVEST (International Standard Randomised Controlled Trial Number: ISRCTN15842444) is an open‐label, non‐randomised, single‐centre, UK‐based, Phase Ib dose escalation, window of opportunity study in patients with bladder UCC undergoing RC. The study will examine two independent treatment routes (one for each route of administration, passive instillation and direct injection, regardless of stage) (see Fig. 1):

- Passive instillation (using a urinary catheter) is the simpler administration route but may not achieve sufficient penetration of atezolizumab into the bladder wall. Passive instillation is the standard route for many NMIBC agents (such as mitomycin, BCG) [22, 23].
- Direct injection of atezolizumab into the bladder tumour and/or surrounding detrusor [24] is also performed under local anaesthesia, is well tolerated while ensuring higher drug penetration into the bladder wall. In the same manner as OnabotulinumtoxinA is currently administered; a long flexible needle is passed down the working channel of a flexible cystoscope. Under direct vision the needle is passed into the bladder wall, and a 0.5 mL bolus is injected.

**Fig. 2:**
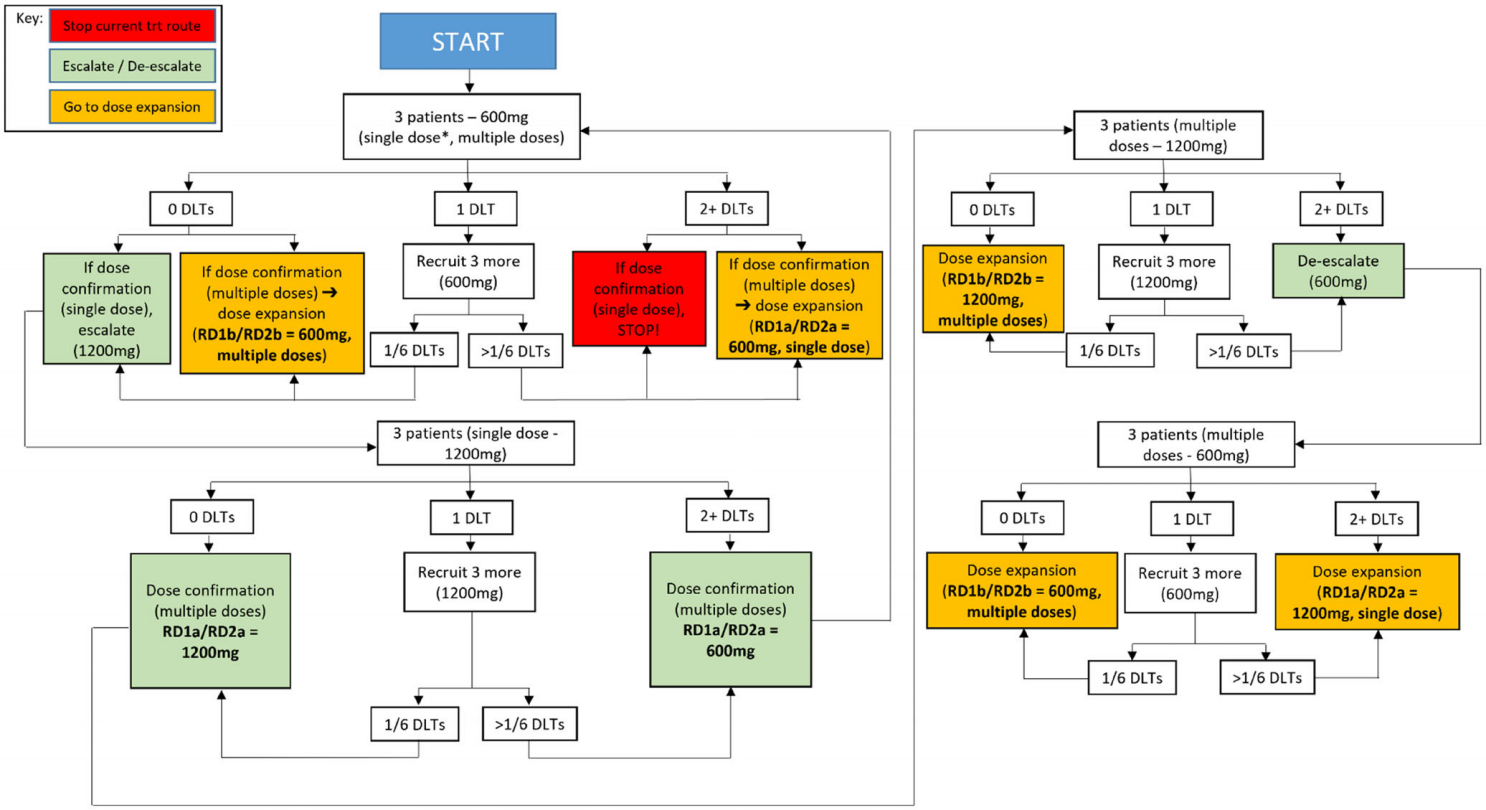
The DLT schema for dose confirmation stages in both treatment routes. * Begin with dose confirmation 1a/2a – single dose. Dose confirmation 1b/2b for multiple doses follows.

For each treatment route, there are dose confirmation stages (single and multiple doses of atezolizumab) using a conventional 3 + 3 design as per Fig. 2, followed by dose expansion stages. Once the recommended dose (RD) is established, a dose expansion stage will further characterise safety and obtain preliminary activity data. A flip‐flop design will allocate participants to whichever treatment route is currently open [25], in order to maximise accrual rates. The direct injection treatment route will be prioritised, at times when both routes are open. This design does not imply a direct comparison.

### Study Aims

We aim to investigate the safety and preliminary activity of intravesical atezolizumab delivered by passive instillation and direct injection into the tumour/bladder wall in patients with bladder UCC prior to RC.

### Endpoints

#### Primary Endpoints

Dose confirmation stages – the number of participants with DLTs from first dose to RC for each confirmation stage: 1a, 1b, 2a and 2b will confirm safety and determine the RD of intravesical atezolizumab.

Dose expansion stages – safety and toxicity will be assessed by the occurrence of AEs, ARs, AEs of special interest (AEoSI), serious AEs (SAEs), serious ARs (SARs) and suspected unexpected serious ARs (SUSARs) (Table 1) graded according to National Cancer Institute (NCI) Common Terminology Criteria for Adverse Events (CTCAE), version 5.0 [26].

**Table 1:** Collection timeframes for safety events within INVEST.

Term	Collection timeframe and applicability
Adverse event (AE)	AEs are collected for all participants that have received trial treatment Reporting begins from initiation of trial treatment until 30 days after RC (or planned RC if no RC takes place) or initiation of new systemic anti‐cancer therapy, whichever occurs first
AEs of special interest (AEoSI)	A specific list of AEoSI in this trial; Cases of potential drug‐induced liver injury that include an elevated ALT or AST in combination with either an elevated bilirubin or clinical jaundice Suspected transmission of an infectious agent by the study treatment Systemic lupus erythematosus Events suggestive of hypersensitivity, infusion‐related reactions, CRS, HLH, and MAS Nephritis Ocular toxicities (e.g., uveitis, retinitis, optic neuritis) Grade ≥2 cardiac disorders (e.g., atrial fibrillation, myocarditis, pericarditis) Vasculitis Autoimmune haemolytic anaemia Severe cutaneous reactions (e.g., Stevens–Johnson syndrome, dermatitis bullous, toxic epidermal necrolysis) AEoSI are collected for all participants that have received trial treatment Reporting begins from initiation of trial treatment until 90 days after RC (or planned RC if no RC takes place) or initiation of new systemic anti‐cancer therapy, whichever occurs first
Adverse reaction (AR)	ARs are collected for all participants that have received trial treatment Reporting begins from initiation of trial treatment until 30 days after RC (or planned RC if no RC takes place) or initiation of new systemic anti‐cancer therapy, whichever occurs first
Serious adverse event (SAE)	After informed consent but prior to initiation of study treatment, only SAEs caused by protocol‐mandated interventions are reported After start of study treatment, all SAEs are reported until 90 days after RC (or planned RC if no RC takes place) or initiation of new systemic anti‐cancer therapy, whichever occurs first
Serious adverse reaction (SAR)	SARs are collected for all participants that have received trial treatment Reporting of SARs begins from initiation of trial treatment until the end of trial
Suspected unexpected serious adverse reaction (SUSAR)	SUSARs are collected for all participants that have received trial treatment Reporting of SUSARs begins from initiation of trial treatment until the end of trial

ALT, alanine aminotransferase; AST, aspartate aminotransferase; CRS, cytokine release syndrome; HLH, haemophagocytic lymphohistiocytosis; MAS, macrophage activation syndrome.

#### Secondary Endpoints

All stages – progression‐free survival (PFS), treatment compliance and pCR rate.

Dose confirmation stages – safety and toxicity will be assessed by the occurrence of AEs, ARs, AEoSI, SAEs, SARs and SUSARs (Table 1) graded according to the NCI CTCAE, version 5.0 [26].

#### Exploratory Endpoints

Overall survival (OS), PK profile (serum and urine concentrations at specified timepoints) and atezolizumab immunogenicity as assessed by baseline prevalence and post‐baseline incidence of anti‐drug antibodies (ADAs) against atezolizumab.

#### Additional Exploratory Objectives

Pre‐ and post‐treatment blood free circulating tumour DNA (ctDNA), peripheral blood mononuclear cells (PBMCs) and serum and urine cell free DNA (cfDNA) are collected to assess drug penetration into the bladder wall and the effects of treatment on circulating tumour cells, local and systemic immune cell composition. Sampling (fresh, frozen, and formalin‐fixed paraffin‐embedded [FFPE] tissue blocks) of scar, tumour and background urothelium is performed on RC specimens. Tissue microarrays will be created from TURBT and RC tissues.

Magnetic resonance imaging (MRI) – fluorodeoxyglucose (FDG) positron emission tomography (PET) (sub‐study); scans are performed prior to and following atezolizumab to non‐invasively assess response of the primary bladder tumour.

### Eligibility

See Table 2 for eligibility criteria.

**Table 2:** Inclusion and exclusion criteria for the INVEST trial.

Inclusion criteria	Exclusion criteria
Patient must be fully informed about the study and have signed the informed consent form	Active UTI in last 2 weeks prior to assessing eligibility
Patients with mixed histologies after TURBT are required to have predominant UCC in the bladder	Severe infection within 4 weeks prior to initiation of study treatment, including, but not limited to, hospitalisation for complications of infection, bacteraemia, or severe pneumonia, or any active infection that could impact patient safety
Patients with pTis to T4N0 M0 for whom RC is planned treatment for their UCC of the bladder. This will include both MIBC and high‐grade NMIBC tumours. For multiple‐dose participants, the time between the planned RC date and confirmation of eligibility must be sufficient to enable participants to receive the minimum required trial treatment (3 weeks) as well as complete the required post‐treatment pre‐RC follow‐up visit (1 week −3/+7 days post‐treatment)	Treatment with therapeutic oral or intravenous antibiotics within 2 weeks prior to initiation of study treatment. Patients receiving prophylactic antibiotics (e.g., to prevent a UTI or COPD exacerbation) are eligible for the study
Patients with MIBC must be ineligible for cisplatin‐based neoadjuvant chemotherapy or have declined neoadjuvant chemotherapy	Presence of indwelling urinary stent or history of vesico‐ureteric reflux
Patient must be aged ≥18 years	Other intravesical therapy within 2 weeks of assessing eligibility
Patient must have ECOG performance status between 0 and 2	Prior radiotherapy to bladder or planned radiotherapy to bladder
Patient must be willing and able to comply with the protocol, have mental capacity and (if woman of childbearing potential) use effective contraception throughout treatment and for 5 months after treatment completion	Reduced bladder capacity (defined as an inability to hold an intravesical instillation for 1 h)
Patient must have diagnostic biopsy tissue material (e.g., TURBT sample) available for use in the trial	Positive pregnancy test, breast‐feeding women, patients intending to become pregnant during and within 5 months after last dose of atezolizumab, or patients unwilling to comply with contraception requirements
Patient must have adequate organ function within 28 days prior to confirmation of eligibility and 7 days of study treatment	Patients with active, known, or suspected autoimmune disease with the following exceptions: Patients with vitiligo or alopecia Patients with hypothyroidism stable on hormone replacement Any chronic skin condition that does not require systemic therapy Patients with coeliac disease controlled by diet alone Patients with Crohn's disease or ulcerative colitis (must be inactive stable disease)
	Treatment with systemic immunosuppressive medication within 2 weeks prior to initiation of study treatment, with the following exceptions: Acute, low‐dose systemic immunosuppressant medication Patients who received mineralocorticoids, inhaled or low‐dose corticosteroids for COPD or asthma, or low‐dose corticosteroids for orthostatic hypotension or adrenal insufficiency
	Positive test for HBV, HCV or HIV
	Patients considered a poor medical risk by the investigator due to a serious, uncontrolled medical disorder, non‐malignant system disease or active uncontrolled infection
	Prior systemic ICI therapy (prior BCG is permitted)
	History of severe allergic anaphylactic reactions to chimeric or humanised antibodies or fusion proteins
	Known hypersensitivity to Chinese hamster ovary cell products
	Prior allogeneic stem cell or solid organ transplantation.
	Treatment with a live, attenuated vaccine within 4 weeks prior to initiation of study treatment or within 5 months after the final dose of atezolizumab
	History of idiopathic pulmonary fibrosis, organising pneumonia, drug‐induced pneumonitis, idiopathic pneumonitis, or evidence of active pneumonitis on CT scan
	Active tuberculosis
	Significant cardiovascular disease within 3 months prior to initiation of study treatment, unstable arrhythmia, or unstable angina
	Major surgical procedure within 4 weeks prior to initiation of study treatment, or anticipation of need for a major surgical procedure during the study (except for RC)
	History of leptomeningeal disease
	Uncontrolled or symptomatic hypercalcemia

COPD, chronic obstructive pulmonary disease; ECOG, Eastern Cooperative Oncology Group HBV, hepatitis B virus; HCV, hepatitis C virus.

## Methods

### Sample Size Determination

Up to 62 evaluable patients will be recruited during the trial.

#### Dose Confirmation Stages

A minimum of six (three by each route) and up to 42 (limit for practical reasons) DLT evaluable participants across both treatment routes will be recruited during the dose confirmation stages. This is based on the number of participants experiencing DLTs within a cohort (*n* = 3) as per the 3 + 3 design (Figs 1 and 2), across four dose confirmation stages (single‐ or multiple‐dose with dose levels 600 and 1200 mg) for each treatment route.

#### Dose Expansion Stages

In all, 10 additional participants for each route (20 in total) will be recruited once a RD has been found from the dose confirmation stages. As no formal hypothesis testing or comparisons will be performed, a powered sample size calculation is not required for this stage.

### Recruitment Process

**Fig. 3:**
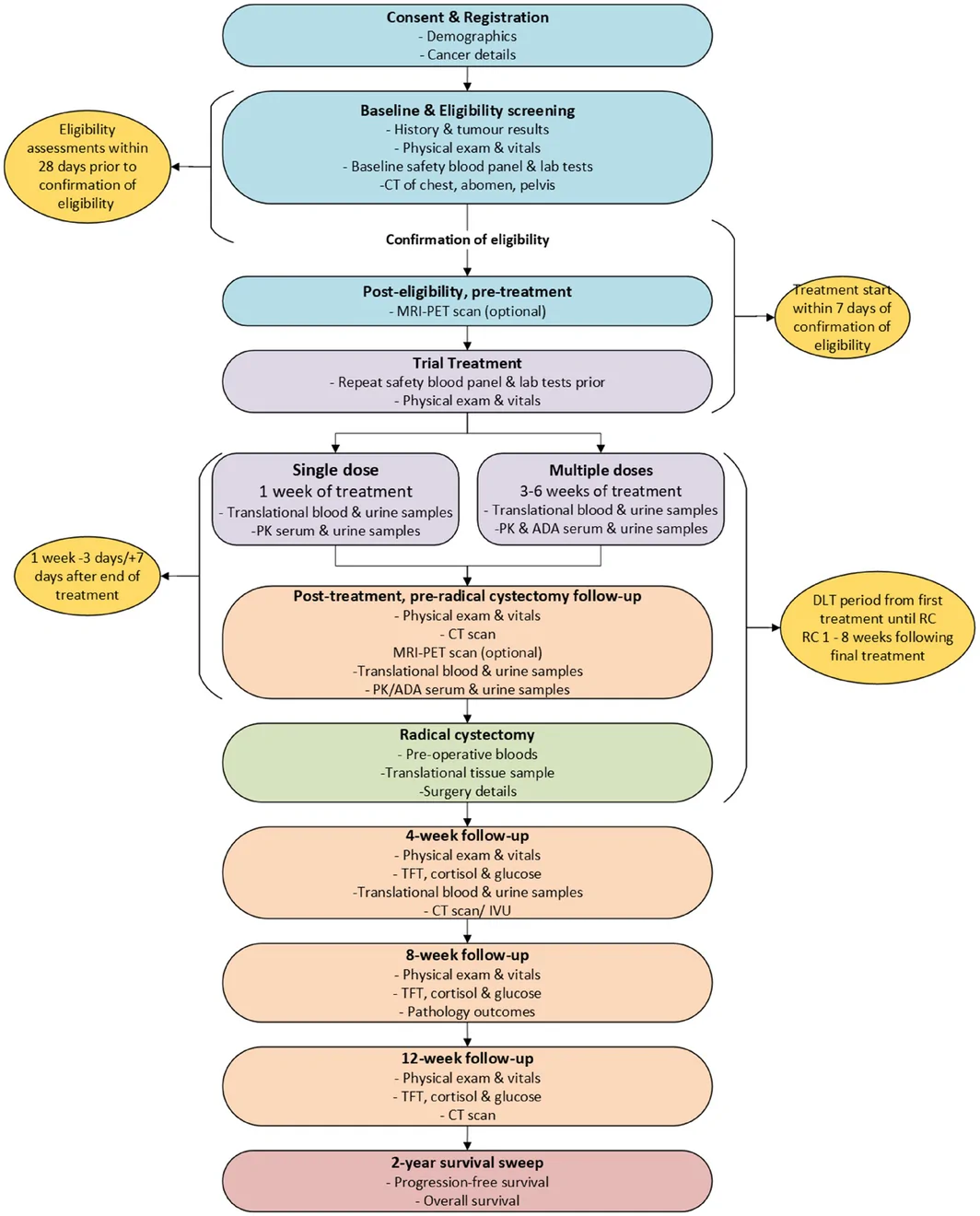
Participant pathway for the INVEST trial.

Participants are recruited from Sheffield Teaching Hospitals NHS Foundation Trust. Other NHS hospitals will be opened if recruitment falls behind the target. Assenting participants are invited to provide informed, written consent to an authorised clinician and are provided with a trial number. Initial demographics and medical history are collected at the time of consent. Participants are then formally assessed for eligibility prior to commencing treatment. Participant flow through the trial is detailed in Fig. 3. The first participant was registered on 11/05/2023 and recruitment is due to continue until March 2026.

### Interventions

Two doses of atezolizumab (600 and 1200 mg) via two routes, are being investigated (Fig. 1). We will start with 600 mg single dose for both routes, and either escalate to 1200 mg single dose or expand to a further three participants depending on the occurrence of DLTs as per the 3 + 3 design (Fig. 2); we will then explore multiple doses. For both routes in the single dose cohorts (dose confirmation stages 1a and 2a), treatment is for 1 week only. In dose confirmation stages 1b and 2b, between 3 and 6 weeks of treatment (one dose per week) depending upon waiting list time and tolerability will take place prior to RC.

For the dose expansion stages, participants will be treated according to the RD identified in the respective dose confirmation stage (Fig. 2). Treatment is given for 3–6 weeks unless multiple dosing was not deemed acceptable in dose confirmation stage 1b/2b, in which case the dose expansion stage will assess single dose treatment as per the schedule for dose confirmation stage 1a/2a.

The final dose of trial treatment must be administered ≥1 week before RC and is recommended to be within 4–8 weeks. If RC is due to take place >8 weeks after final trial treatment, the decision will rest with the Safety Review Committee (SRC) whether to replace the participant.

#### Routes of Administration

Treatment route 1 – passive instillation: the bladder is filled with atezolizumab via a urethral catheter (flushed with normal saline). Atezolizumab is left in place for ~1 h, before voiding.

Treatment route 2 – direct injection: under local anaesthetic, a flexible cystoscope is passed into the bladder. Atezolizumab (20 mL in volume) is directly injected into the tumours and adjacent bladder wall in 0.5–1.0 mL boluses. Participants are observed within the department for 1 h to include voiding urine.

#### Criteria for Dose Discontinuation/Modifications

Participants receiving multiple doses (dose confirmation 1b and 2b; dose expansion 1 and 2 if applicable) are assessed prior to each treatment (including blood tests with thresholds which must be met), to determine whether they can continue. In the event of a DLT, trial treatment will be permanently discontinued. There are also immune‐mediated toxicities, for which guidance is given to investigators to pause or withdraw treatment depending on the toxicity and grade.

#### The DLTs

Assessed from Day 1 of trial treatment until RC (or 4 weeks after final dose if RC does not occur), DLTs are defined as any of the following events if deemed to be related to atezolizumab by the SRC:

- A treatment‐related toxicity that delays RC by >2 weeks.
- Grade ≥3 haematuria.
- Any Grade ≥3 immune‐mediated toxicity.
- Any SAR.
- Grade 4 haematological or Grade ≥3 non‐haematological toxicity considered treatment‐related.
- Any other treatment‐related event decided by the SRC to be clinically significant.

Events considered by the SRC to be highly unlikely to be related to atezolizumab will not be categorised as a DLT.

### Assessments

Figure 3 details the participant pathway in the INVEST study. Participants are assessed at baseline, prior to each trial treatment (for a maximum of 6 weeks), and at 1 week following their final trial treatment. All participants are expected to undergo RC within 1–8 weeks of their final trial treatment (as per [27]), and follow‐up visits will take place 4, 8 and 12 weeks after the date of RC. Assessments include CT of chest, abdomen and pelvis (at baseline, 1 week after final trial treatment, at the 12‐week follow‐up visit, and if clinically indicated), and an optional MRI‐FDG PET scan of the bladder (prior to the start of trial treatment and at 1 week after final trial treatment).

Serum and urine samples for atezolizumab PK are taken immediately prior to and after each dose of trial treatment. On Week 1 of treatment, additional samples are taken after the dose is administered, with four samples of each taken across 6 h. Serum samples for ADA analysis are only taken for participants receiving multiple doses, prior to treatment on Weeks 1, 3 and 6. Serum and urine samples are also collected 1 week after final trial treatment. Collection, storage and processing of samples is detailed in a separate sample management standard operating procedure.

The TURBT and RC tissue are assessed as per the Royal College of Pathologists Dataset for histopathological reporting of tumours of the urinary collecting system [28]. Tissue samples for translational analysis are collected from diagnostic biopsy tissue at baseline, and from bladder tissue at RC. Whole blood, PBMCs, serum, plasma and urine samples for translational work are taken on Week 1, with PBMCs, serum and urine taken prior to each subsequent week of treatment for multiple dose participants. PBMCs, serum, plasma and urine are also taken at 1 week after final trial treatment. This work will take the form of a separate research project which will be subject to separate ethical and regulatory review and approval as appropriate. Participation is optional and requires participants to opt‐in at time of consent.

Once all post‐RC follow‐up visits have been completed, formal trial surveillance will cease. A data sweep will be completed 2 years after registration to collect PFS and OS data. Details of any SARs and SUSARs are collected until the end of the trial.

### Methods of Data Collection and Management

Investigations in this trial use the results of local assessments in conjunction with trial‐specific assessments. Data are collected by research staff at sites via electronic data capture using the MACRO database, which is centrally managed by the Clinical Trials Research Unit (CTRU), University of Leeds. CTRU will conduct source data verification checking from recruitment until the end of the DLT period and will control the final trial datasets. All information collected during the course of the trial will be kept strictly confidential. Information will be held securely on paper and electronically at the CTRU and research site(s). During the trial, participant data will be pseudo‐anonymised and coded with a trial number rather than participant names, and will include two participant identifiers, usually the participant's initials and date of birth. Following the end of the trial, any data shared with other researchers will be anonymised and individual participants will not be identifiable. The CTRU will comply with all aspects of the Data Protection Legislation including General Data Protection Regulation (Regulation [EU] 2016/679), the Data Protection Act 2018, and any successor legislation.

#### Independent Data Monitoring

The SRC, consisting of three independent clinical members and one patient and public involvement and engagement (PPIE) representative, meets regularly to review the safety and ethics of the study by regularly reviewing the safety data.

During the dose confirmation stages, the first single dose and multiple dose participants in each treatment route are recruited and observed for the DLT reporting period, prior to the SRC confirming any further recruitment. After all participants in a cohort have completed their DLT period, the CTRU trial statistician produces a report with a summary of each individual participant, including their DLTs, detailed safety listings, treatment data and RC data. The SRC will meet to assess this report and to confirm the dose the next cohort of participants will receive within the respective treatment route.

### Analysis Plan

No formal analyses are planned until after the trial has closed to recruitment. Final analysis, except from PFS and OS, will be conducted after all participants in the study have been followed up for at least 6 months after registration. PFS and OS analyses will be conducted once all participants have either had data collected in the follow‐up sweep 2 years after the registration date of the final participant, or once all participants have recorded disease progression or death as appropriate to the analysis, whichever is sooner. Exploratory sub‐analyses will include breakdown by for example stage or phenotype. See Appendix S1 for the full plan.

#### Analysis Populations

All analysis populations will be split by treatment route, and if applicable dose level.

- Dose confirmation DLT evaluable population: participants who receive ≥80% of the assigned atezolizumab dose in the single dose confirmation stages and ≥80% of the total of three times the assigned atezolizumab dose over ≥3 treatment weeks in the multiple dose confirmation stages. Participants who are not evaluable for DLTs and do not experience a DLT are replaced.
- Dose confirmation safety population: dose confirmation stage participants who receive one or more dose(s) of atezolizumab.
- Dose expansion safety population: participants who receive one or more dose(s) of atezolizumab within the dose expansion stage or are treated at the RD within the dose confirmation stage.
- Dose expansion analysis population: participants who receive one or more dose(s) of atezolizumab within the dose expansion stage or are treated at the RD within the dose confirmation stage. This is equivalent to the dose expansion safety population.

#### Endpoint Analyses

The primary, secondary and exploratory endpoint analyses are outlined in Table 3. Full details of the additional exploratory translational endpoints will be specified in a separate Translational Science Protocol.

**Table 3:** Endpoint analyses for the INVEST trial.

Endpoint	Stage of trial	Analysis population	Details of analysis methods
**Primary endpoints**			
DLTs	Dose confirmation stages	Dose confirmation DLT evaluable populations	Number of participants experiencing DLTs from Day 1 of trial treatment until RC (or 4 weeks after final dose if RC does not occur) Descriptive summaries of the specific DLT events observed
Safety and toxicity	Dose expansion stages	Dose expansion safety populations	Number of SAEs (umbrella term that includes SARs and SUSARs) Proportion of participants experiencing at least one SAE and number of SAEs experienced per participant. Descriptive summaries of SAEs according to relationship to trial treatment, seriousness criteria, causality, MedDRA body system code, expectedness and outcome Proportion of participants experiencing the maximum grade of each toxicity Descriptive summaries for relatedness of AEs and AEoSI to treatment alone and/or RC All AEs and AEoSI will be tabulated If appropriate, events will be summarised by timing
**Secondary endpoints**			
PFS	All stages	Dose confirmation DLT evaluable populations; dose expansion analysis populations	PFS curves calculated using the Kaplan–Meier method Median PFS estimates and PFS estimates at 3‐, 6‐, 12‐ and 24‐ months
Treatment compliance (including compliance with RC)	All stages	Dose confirmation safety populations; dose expansion safety populations	Descriptive summaries for dose delays, dose reductions, dose omissions and withdrawals, including reasons Number of participants with delays to RC or who do not undergo RC, including reasons Multiple dose participants only: number of doses received, number of doses originally planned and reasons for stopping treatment Direct injection participants only: descriptive summary of volume of atezolizumab injected into the tumour/bladder wall
pCR rate	All stages	Dose confirmation DLT evaluable populations; dose expansion analysis populations	Proportion of participants experiencing pCR according to the local pathology report post‐RC If appropriate, corresponding 95% CIs will be calculated
Safety and toxicity	Dose confirmation stages	Dose confirmation safety populations	Assessed as per the dose expansion primary endpoint of safety and toxicity, presented by treatment route and dose level
**Exploratory endpoints**			
OS	All stages	Dose confirmation DLT evaluable populations; dose expansion analysis populations	OS curves calculated using the Kaplan–Meier method Median OS estimates and OS estimates at 6‐, 12‐ and 24‐ months Tabular summary of causes of participant deaths
PK profile as assessed by serum and urine concentrations of atezolizumab at specified time points	All stages	Dose confirmation DLT evaluable populations; dose expansion analysis populations	Plots for concentration–time data for each participant over time May include but not necessarily be limited to the following PK parameters: maximum plasma concentration; plasma half‐life; time to maximum plasma concentration; area under the concentration time curve
Atezolizumab immunogenicity as assessed by baseline prevalence and post‐baseline incidence of ADAs against atezolizumab	All stages	Dose confirmation DLT evaluable populations; dose expansion analysis populations	Multiple dose participants only: Number and proportion of ADA‐positive and ADA‐negative participants, at baseline and after the start of trial treatment

MedDRA, Medical Dictionary for Regulatory Activities.

### Study Organisation and Administration

The INVEST study is sponsored by Sheffield Teaching Hospitals NHS Foundation Trust. Additional support is also provided by the National Institute for Health Research through the use of the Clinical Research Network. Trial supervision is provided by Leeds CTRU, who will provide set‐up and monitoring of trial conduct in line with CTRU Standard Operating procedures and Good Clinical Practice Conditions and Principles as detailed in the UK Medicines for Human Use (Clinical Trials) Regulations 2004 and UK Policy Framework for Health and Social Care Research 2017. Three PPIE representatives are involved in the study as members of the Trial Management Group (TMG). This group is responsible for the ongoing management of the trial and meet frequently to discuss the ongoing status of the study and will be involved in decisions on plans regarding dissemination of the research. These PPIE representatives are also involved in the development of the participant information sheet (see Appendix S2) and informed consent document to ensure it is informative and meets the information needs of the potential participants.

When the study is complete the results will be published in a medical journal, but no individual participants will be identified. Requests for access to anonymised datasets will be considered after results have been reported by contacting ctru-dataaccess@leeds.ac.uk. The participant information sheet (Appendix S2) explains that should a participant wish to see the published results they are advised to contact the investigator/research staff at their hospital. Plans for dissemination will be discussed with PPIE representatives on the TMG and the SRC. We plan to issue a thank you letter to participants with a lay summary of the results.

A Standard Protocol Items: Recommendations for Interventional Trials Dose‐finding Extension Checklist (SPIRIT‐DEFINE) has been prepared for this manuscript (see Appendix S3) [29].

The INVEST trial is registered (ISRCTN15842444 and European Union Drug Regulating Authorities Clinical Trials [EudraCT] number 2021–006537‐19) and received initial Research Ethics Committee approval on 8 November 2023 and initial Medicines and Healthcare products Regulatory Agency (MHRA) approval on 9 November 2023. The current protocol is version 5.0 dated 5 December 2024, with updates shared on the ISRCTN registry.

## Discussion

The ICIs have changed the landscape of patients with advanced BCa. Recent advances in HRNMIBC have raised the possibility of their use in earlier disease settings [30]. ICIs are generally well tolerated, although immune‐mediated toxicity may lead to systemic side effects to lungs, kidneys, heart, skin, endocrine glands, gastrointestinal systems that can manifest in acute and long‐term toxicities. By delivering this treatment intravesically, it has a potential to reduce the systemic side effects while improving disease control in this indication. To understand the safety of this agent via the intravesical route, we are comparing both passive instillation and direct injection into the bladder tumour/wall. Passive instillation (using a urinary catheter) is the simpler administration route but may not achieve sufficient penetration of atezolizumab into the bladder wall. Passive instillation is the standard route for many NMIBC agents (such as mitomycin, BCG) and most units have nursing care pathways for outpatient delivery [19, 20]. Direct injection of atezolizumab into the bladder tumour and/or surrounding detrusor [21] is also performed under local anaesthesia, is well tolerated, and known to be safe, while ensuring higher drug penetration into the bladder wall. Translational samples including pre‐ and post‐treatment blood ctDNA, PBMCs and serum and urine cfDNA are being collected to assess drug penetration into the bladder wall and the effects of treatment on circulating tumour cells, local and systemic immune cell composition. Sampling (fresh, frozen, and FFPE blocks) of scar, tumour and background urothelium is performed on RC specimens. Tissue microarrays will be created from TURBT and RC tissue. An MRI‐PET sub‐study is being undertaken, with scans prior to and following the trial immunotherapy to assess the response of the primary bladder tumour(s) to this treatment.

While the study is not assessing efficacy as a primary endpoint, collection of samples and MRI‐PET scans will provide insights into potential effects of atezolizumab on tumour and non‐tumour cells.

As this is a single‐centre study, the generalisability of the results may be limited, but will help inform a larger multicentre trial. However, this is sufficient for an initial study of feasibility and safety, and any further trials will be designed as multicentre trials with larger participant populations.

The results of this Phase Ib window of opportunity study will help inform the design of subsequent studies to explore the potential benefits of these targeted and less invasive treatments to this patient population. If the INVEST study confirms safety, further Phase II multicentre randomised studies to confirm efficacy, followed by randomised multicentre Phase III trials to confirm if a subset of patients may be spared of RC and preserve their bladder with improvements in recurrence‐free survival with intravesical use of atezolizumab treatment.

## Author Contributions

Syed A. Hussain, James W. F. Catto, Jessica Kendall, Jamie B. Oughton and FC designed the study. RLW, Jessica Kendall, Jamie B. Oughton, JG, FC, James W. F. Catto, Syed A. Hussain, JG, SK, JT, and RH contributed to the development of the protocol. Jessica Kendall and ES developed the statistical analysis plan and are responsible for the ongoing statistical monitoring, analysis and interpretation of the data. RH, SK, AN, DJR, James W. F. Catto and Syed A. Hussain perform the research and collect data. JG performs the translational research and collects data. RH and SK provide imaging support and advice. RLW and JBO perform trial management. RLW, ES, Jessica Kendall, Jamie B. Oughton, James W. F. Catto and Syed A. Hussain drafted the manuscript with critical input from all other authors, who reviewed, read and approved the final manuscript. The manuscript was written based on version 5.0 of the protocol (05/12/2024).

## Funding

This trial is funded by F. Hoffmann‐La Roche Ltd. The research is supported by F. Hoffmann‐La Roche Ltd. provided funding to Sheffield Teaching Hospitals NHS Foundation Trust, who in turn is the sponsor for the INVEST trial. Roche approved the design of the study but have no input in the collection, analysis or interpretation of the data as this is an NHS sponsored trial. Roche are also providing the study drug (atezolizumab) for the trial. The views expressed are those of the author(s) and not necessarily those of the NHS, the UK National Institute for Health Research (NIHR) or the Department of Health.

## Ethics Approval

This study is conducted in accordance with Good Clinical Practice guidelines and the principles of the Declaration of Helsinki. It has been approved by London ‐ Central Research Ethics Committee (ref approval number 22/LO/0651). Participants provide written informed consent before trial related procedures.

## Disclosure of Interests

Ruby Lister‐Whelan has received research funding from Roche, AstraZeneca and the NIHR. Ethan Senior has received research funding from Roche, Takeda, Celgene, Merck, Amgen and Sanofi. Jessica Kendall has received research funding from Roche, AstraZeneca, Cancer Research UK, NIHR and Karyopharm. Jamie B. Oughton has received research funding from Roche, AstraZeneca, Cancer Research UK and the NIHR. Fiona Collinson has received research funding from Roche, AstraZeneca, Cancer Research UK and the NIHR. Jon Griffin has received research funding from Roche and in‐kind research support from Owkin; and has received honoraria from ISSECAM. Steven Kennish has received support for attending meetings from Bracco. Derek J. Rosario has received research funding from Boston Scientific and holds the position of President of the Urostomy Association. Irbaz B. Riaz has received research funding from the American Society of Clinical Oncology. Julie Telliez has stock options in F. Hoffman‐La Roche Ltd. and is employed by them. James W. F. Catto has received and research funding from Roche; has received consulting fees from AstraZeneca, Bristol Myers Squibb, Gilead, QED Therapeutics, Roche, Ferring, Steba Biotech, UroGen, Janssen, Photocure and Pfizer; has received speaker fees or honoraria from Bristol Myers Squibb, AsztraZeneca, Roche and Medscape; has participated in advisory boards for the PROMOTE and CISTO trials and has a trustee role in Fight Bladder Cancer UK and Weston Park Cancer Charity. Syed A. Hussain has received research funding from Cancer Research UK, MRC/NIHR, UHB charities, CCC charities, North‐West Cancer Research, Yorkshire Cancer Research, Weston Park Cancer Charity, Bayer, Janssen, Boehringer Ingelheim, Pierre Fabre, Eli Lilly, Roche; and has received advisory board/consultancy fees from Roche, MSD, AstraZeneca, BMS, Janssen, GSK, Astellas, Pfizer, Bayer, Merck, Pierre Fabre, Sotio, Gilead. Rachel Hubbard and Aidan Noon have no competing interests to declare.

## Trial Status

Protocol: version 5.0, 5 December 2024. Date opened to recruitment: 04/05/2023. Expected recruitment closure: 31/08/2026. Data collection will continue until 31/08/2028.

## Supporting information


**Appendix S1.** Statistical analysis plan.

**Appendix S2.** Participant information sheet.

**Appendix S3.** The SPIRIT‐DEFINE downloadable checklist.

## Data Availability

Data from trial will be made available following publication of the results upon reasonable request subject to study contracts and approval of SRC or TMG.
