# Supplementary material for: INtraVESical immunoTherapy (INVEST) prior to radical cystectomy for bladder cancer: trial protocol
Source: BJU Int. 2026 Jul 27;138(4):607–19. doi: 10.1111/bju.70387 (PMC13550807; doi:10.1111/bju.70387)
Supplement: Supplementary file 1 [file BJU-138-607-s002.pdf]

CLINICAL TRIALS RESEARCH UNIT (CTRU)
UNIVERSITY OF LEEDS

FINAL
STATISTICAL ANALYSIS PLAN

INVEST

VERSION 2.0

JUNE 2024

Trial Statistician:	Ethan Senior
Supervising Statistician:	Jessica Kendall
Trial Management Lead:	Jamie Oughton
Senior Trial Co-ordinator:	Ruby Smith Whelan
Data Manager:	Dominic McCready
CTRU Scientific Lead:	Dr. Fiona Collinson
Chief Investigator:	Prof. Syed Hussain

Table of Contents

1 Introduction ... 3
1.1 Background ... 3
1.1.1 Rationale for intravesical study design ... 3
1.1.2 Route of administration ... 4
1.1.3 Clinical hypotheses ... 4
1.2 Design ... 4
1.3 Allocation of participants to treatment routes ... 5
1.4 Aims ... 5
1.4.1 Primary objectives ... 5
1.4.2 Secondary objectives ... 5
1.4.3 Exploratory objectives ... 5
1.5 Sample size and expected accrual ... 5
1.6 Planned analyses ... 6
2 Endpoints ... 6
2.1 Primary endpoint ... 6
2.2 Secondary endpoints ... 7
2.2.1 All stages ... 7
2.2.2 Dose confirmation stages ... 7
2.3 Exploratory endpoints ... 7
2.4 Derivation of endpoints ... 7
2.4.1 DLTs ... 7
2.4.2 Safety and toxicity ... 7
2.4.3 Other secondary and exploratory endpoints ... 8
2.5 Missing data ... 8
3 Populations ... 8
3.1 Eligibility ... 8
3.2 Dose confirmation stages ... 8
3.3 Dose expansion stages ... 9
4 Data Handling ... 9
4.1 Data monitoring ... 9
4.2 SRC ... 10
4.3 Data validation ... 10
4.4 Procedures ... 11
5 Data Analysis ... 11
5.1 General calculations ... 11
5.2 Statistical Considerations ... 11
5.2.1 Participant replacement ... 11
5.3 Analysis ... 11
5.3.1 Study summary ... 11
5.3.2 Baseline characteristics ... 12
5.3.3 Primary endpoint analysis ... 12
5.3.4 Secondary endpoint analysis ... 13
5.3.5 Exploratory endpoint analysis ... 14
6 Reporting and Dissemination of the Results ... 15
7 References ... 16
8 Appendices ... 17
8.1 Appendix 1 – Trial flow diagram ... 17
8.2 Appendix 2 – DLT schema for both treatment routes ... 18
8.3 Appendix 3 – RECIST 1.1 guideline ... 18
8.4 Appendix 4 - Approval of Analysis Plan ... 19

1 Introduction

This statistical analysis plan has been written using the INVEST Protocol V3.0.

1.1 Background

Bladder cancer is the third most prevalent cancer and one of the most expensive to treat [1, 2]. Bladder tumours are mostly urothelial cell carcinoma (UCC) in histological type and stratified into non-muscle invasive bladder cancers (NMIBC) and muscle invasive bladder cancers (MIBC) according to stage. NMIBC is currently managed by combinations of local surgical resection, intravesical chemotherapy, intravesical immunotherapy (e.g. Bacillus Calmette–Guérin [BCG]) and regular surveillance [3, 4]. Most patients develop multiple bladder tumours (local recurrence in up to 75%) and a minority (up to 25%) develop progression to muscle invasion, metastases and death (10-15%) [5, 6]. Current treatments are laborious, poorly tolerated, have supply limitations and fail to control the disease in many patients [7]. As such, novel treatments are needed to improve the outcomes of patients with NMIBC. The specific main goals of novel agents would be to have lower toxicity, improved efficacy (i.e. reduce the risk of progression) and a more sustainable manufacturing program than BCG. Useful, but less important, goals would also be the ability to add multiple agents into combined regimens and to combine local and systemic approaches.

Intravesical immunotherapy has been used for many years to treat patients with NMIBC. As such, this is an immune-responsive disease and so we wish to explore the use of more advanced immune treatments. We believe that intravesical immune checkpoint inhibition (ICI) may offer such an option for NMIBC.

Atezolizumab is an immune checkpoint inhibitor which targets Programmed death-ligand 1 (PD-L1). Various observations suggest atezolizumab may reach the area of tumour/T cell interaction if passively instilled intravesically or injected directly into the tumour/bladder wall. Given that support for our hypothesis can be derived from murine bladder cancer models testing local and systemic ICI [8], and the possibility that intravesical atezolizumab can access the tumour/T cell domain in NMIBC, we need to understand the safety of this agent (when delivered intravesically) and whether it is feasible to use this agent within this clinical setting.

Atezolizumab has been dosed via intravenous (IV) route for various cancer indications. With regards to bladder cancer, survival in patients with inoperable advanced urothelial cancer ineligible for cisplatin-based chemotherapy is in the range of 8-9 months. With the use of atezolizumab in this patient population group the survival is consistently in the range of 12-18 months. IMvigor 210 showed the survival to be 15.9 months [9, 10].

IMvigor 211 randomly assigned 931 patients to receive atezolizumab (n=467) or chemotherapy (n=464). Atezolizumab was not associated with significantly longer overall survival (OS) than chemotherapy in patients with platinum-refractory metastatic urothelial carcinoma overexpressing PD-L1 (IC2/3). However, the safety profile for atezolizumab was favourable compared with chemotherapy. Exploratory analysis of the intention-to-treat population showed well-tolerated, durable responses in line with previous phase II data for atezolizumab in this setting [11].

1.1.1 Rationale for intravesical study design

Overall, the current data support the intravesical route of administration assuming a good in vitro vs. in vivo correlation, healthy vs. bladder cancer translatability of urine stability. It is recommended to measure the baseline pH of patient urine [12].

Previous researchers have commenced similar work using intravesical ICIs manufactured by other companies. These include the PemBla study, from Oxford, using pembrolizumab instillations [13]; the SUBDUE-1 study, from Perth, Australia, using durvalumab sub-urothelial injections [14]; the NCT03759496 study through a Greek study group [15] using durvalumab instillations and the North American NCT02808143 study of intravesical pembrolizumab and BCG in North America [16].

Given this novel route for administration, the safest, most tolerable, most active dose for each agent is unknown. For passive instillation, the PemBla study group are exploring doses between 50-200mg pembrolizumab. The NCT03759496 group are using doses for instillation within the 500-1000mg range of durvalumab. For direct injection, the SUBDUE-1 study team are using 3 doses of durvalumab (25, 75 and 150mg). Within this trial, we will explore 600 and 1200mg atezolizumab, reflecting prior researchers' proposals, the volumes of agents needed to fill the bladder, a pragmatic choice around drug delivery and considering that

the bladder will be removed shortly. Both routes of administration (outlined in Section 1.1.2) will start with the lower dose (600mg), and for the direct injection route we will record how much of the agent can be injected into the tumour/adjacent bladder wall.

The majority of patients in clinical practice received 1200mg IV of atezolizumab administered every three weeks (q3w). In this trial we will use either 600mg or 1200mg up to once per week (q1w) in the multiple dose stage. The rationale for giving weekly intravesical or intratumoural dose compared to q3w intravenous dose is because we anticipate significantly less absorption via passive bladder instillation and intratumoural routes of administration, when compared with intravenous administration of atezolizumab, and so we expect fewer side effects and fewer problems with accumulation of drug effects, as supported by data from the PemBla study.

1.1.2 Route of administration

i. Passive instillation

Passive instillation is the easiest administration route, using a urinary catheter, but may not achieve sufficient penetration of atezolizumab into the bladder wall. Passive instillation is the standard route for many bladder cancer agents (such as mitomycin, BCG, docetaxel, gemcitabine and epirubicin). Most units have nursing care pathways for outpatient delivery. The risks and adverse event profile from passive installation of standard drugs/BCG are well known [17, 18].

ii. Direct injection

An alternate route, as used in the clinic for Botox administration into the detrusor, is direct injection [19]. This is also performed under local anaesthesia, is well tolerated and known to be safe, but ensures higher drug penetration into the bladder wall.

1.1.3 Clinical hypotheses

Intravesical atezolizumab (either passive instillation or direct injection) has low toxicity, is well tolerated by patients, and penetrates the bladder wall to reach the tumour/T cell interface.

1.2 Design

INVEST is an open-label, non-randomised, phase Ib window of opportunity study in patients that are undergoing radical cystectomy (RC) for bladder cancer (high grade stages pTis to T4 N0 M0).

The overall trial design is summarised in the trial flow diagram in Figure 1. The study will examine two treatment routes (one for each of the two routes of administration as outlined in Section 1.1.2 – passive instillation and direct injection). These treatment routes are completely independent of each other and will run in parallel. Patients will be allocated to a treatment route based on cohort availability and local logistics (see Section **Error! Reference source not found.**). Within each treatment route, the trial will be composed of two sequential dose confirmation stages (single dose of atezolizumab, then multiple doses of atezolizumab), followed by an expansion stage.

The dose confirmation stages use a conventional 3+3 design to establish the safety of both passive instillation of intravesical atezolizumab (dose confirmation 1a: single dose, followed by dose confirmation 1b: multiple dose) and direct injection of intravesical atezolizumab (dose confirmation 2a: single dose, followed by dose confirmation 2b: multiple dose). The dose of atezolizumab administered to each cohort will be determined based upon the number of dose-limiting toxicities (DLTs) experienced within the preceding cohort (Figure 2). The treatment routes are not dependent upon each other for proceeding to each stage.

During the dose confirmation stages, recruitment to a treatment route will be paused to allow safety to be evaluated within that respective route (see Section 1.6). To optimise recruitment, the flip-flop (or ping-pong) design [20] will be utilised such that when one treatment route is closed to recruitment, the other treatment route will then be opened to recruitment. This will ensure that eligible patients presenting during the dose confirmation stages are able to be recruited to one of the two treatment routes, maximising the number of patients recruited overall.

Once the recommended dose (RD) has been established within dose confirmation stage 1b or 2b, the respective expansion stage will open to further characterise the safety profile and preliminary information on activity. If we are not able to establish a RD for multiple dosing (RD1b/RD2b) we will evaluate the RD1a/RD2a

single dose in the respective expansion stage.

The final analysis will take place after all participants have been followed up for a minimum of 6 months post-registration. Progression-free survival (PFS) and OS will be reported to provide preliminary information on efficacy. For PFS and OS, data collected in the follow-up sweep 2 years after the registration date of the final participant will be used, unless a progression and/or death (as appropriate) is recorded sooner.

1.3 Allocation of participants to treatment routes

To minimise the potential for selection bias in the unlikely situation where both treatment routes have patient slots available and local logistics allow for recruitment to both treatment routes, then treatment route 2 (direct injection) will be prioritised, as logistics may not always allow for recruitment into this route. The exception to this situation is when a DLT has occurred at the current dose level within treatment route 1 (passive instillation). The Safety Review Committee (SRC) may overrule the pre-specified prioritisation schedule in advance of approaching individual participants.

1.4 Aims

1.4.1 Primary objectives

<u>Dose confirmation stages:</u>

- To determine the toxicity and recommended dose (RD) for further investigation of atezolizumab by passively instilled intravesical administration.
- To determine the toxicity and RD for further investigation of atezolizumab when injected directly into the tumour/bladder wall via the intravesical route.

<u>Dose expansion stages:</u>

- To evaluate the safety and toxicity of passively instilled intravesical atezolizumab at the RD.
- To evaluate the safety and toxicity of directly injected atezolizumab into the tumour/bladder wall at the RD.

1.4.2 Secondary objectives

For each route of atezolizumab administration (passively instilled atezolizumab and directly injected atezolizumab into the tumour/bladder wall) the secondary objectives are:

<u>All stages:</u>

- To estimate PFS
- To assess treatment compliance
- To assess pathological complete response (pCR) rate

<u>Dose confirmation stages:</u>

- To assess safety and toxicity.

1.4.3 Exploratory objectives

- To estimate OS
- To assess the pharmacokinetics (PK) profile of atezolizumab by evaluating serum and urine concentrations
- To assess baseline prevalence and post-baseline incidence of ADAs (anti-drug antibodies) to atezolizumab
- To obtain biological specimens for further translational analyses.

1.5 Sample size and expected accrual

Up to 62 evaluable participants pre-RC are expected to be recruited across the dose confirmation and dose expansion stages of the trial. This includes a minimum of 6 and a maximum of 42 across the dose confirmation

stages, and a further 20 (10 per treatment route) in the dose expansion stages (see **Error! Reference source not found.**).

Dose confirmation stages

Within each parallel treatment route (passive instillation and direct injection), the trial will be composed of two sequential dose confirmation stages (single dose of atezolizumab, then multiple doses of atezolizumab) employing a 3+3 design, with each confirmation stage investigating a maximum of 2 doses (600mg and 1200mg).

A minimum of 3 and a maximum of 12 DLT-evaluable participants will be needed for each of the dose confirmation stages. However, for practical reasons this will be limited to a maximum of 42 DLT-evaluable participants across all dose confirmation stages. The exact sample size will depend upon patient evaluability, the number of DLTs, the severity of toxicities experienced, and will ultimately be determined by the SRC.

Participants receiving insufficient treatment to be evaluated for DLTs (as described in Section 5.2.1*)*, and who do not experience a DLT (defined in Section **Error! Reference source not found.**), will be replaced at the current dose level. This number is expected to be minimal.

Dose expansion stages

At the RD identified during the second dose confirmation stage (multiple doses), a further 10 participants will be recruited within each treatment route to the dose expansion stage.

No formal hypothesis testing or comparisons between treatment routes will be conducted in the dose expansion stage, so a powered sample size calculation is not required.

1.6 Planned analyses

During the dose confirmation stages, the first single dose and multiple dose participants in each treatment route will be recruited and observed for the DLT reporting period, prior to any further participants being recruited to that respective first cohort. On these occasions, the SRC will then meet to assess the safety data, and following SRC approval, a further 2 evaluable participants will be recruited to that respective first cohort. Following these initial first cohorts, recruitment to the respective treatment route will be halted until after each cohort of 3 participants have been observed for the DLT reporting period. The SRC will then meet to assess the safety data and to confirm the dose for the next cohort of participants within that treatment route. CTRU will produce a summary of DLTs at each review point, detailed safety listings and recruitment information for consideration by the SRC. SRC meetings can be convened at an earlier time point if warranted.

Frequency of SRC meetings during the dose expansion stage will be decided by the SRC.

No formal analyses are planned until after the trial is closed to recruitment and the required number of participants have been recruited. Final analysis will be conducted when the required number of participants have been recruited and all participants have been followed up for at least 6 months post-registration. The PFS and OS analyses will be conducted when the required number of participants have been recruited and all participants have either had data collected in the follow-up sweep 2 years after the registration date of the final participant or have recorded progression and/or death (as appropriate), whichever is sooner.

2 Endpoints

2.1 Primary endpoint

Dose confirmation stages

The primary endpoint for the dose confirmation stages is the number of DLTs (defined in Section **Error! Reference source not found.**) observed during the DLT period (from day 1 of trial treatment up to but not including the time of RC, or 4 weeks after last dose of atezolizumab if RC does not occur) for each dose confirmation stage (1a, 1b, 2a and 2b). This will confirm the safety of passively instilled intravesical atezolizumab (single and multiple dose) and of atezolizumab (single and multiple dose) when injected directly into a tumour/bladder wall.

Dose expansion stages

For the dose expansion stages, safety and toxicity will be assessed by the occurrence of adverse events (AEs), adverse reactions (ARs), adverse events of special interest (AEoSIs), serious adverse events (SAEs), serious adverse reactions (SARs) and suspected unexpected serious adverse reactions (SUSARs) graded according to National Cancer Institute (NCI) Common Terminology Criteria for Adverse Events (CTCAE) Version 5.0.

2.2 Secondary endpoints

For each route of atezolizumab administration (passively instilled intravesical atezolizumab and directly injected atezolizumab into the tumour/bladder wall) the secondary endpoints are:

2.2.1 All stages

- PFS
- Treatment compliance
- pCR rate

2.2.2 Dose confirmation stages

Safety and toxicity will be assessed by the occurrence of AEs, ARs, AEoSIs, SAEs, SARs and SUSARs graded according to NCI CTCAE Version 5.0.

2.3 Exploratory endpoints

- OS
- PK profile as assessed by serum and urine concentrations of atezolizumab at specified time points
- Atezolizumab immunogenicity as assessed by baseline prevalence and post-baseline incidence of ADAs against atezolizumab

Full details of the exploratory translational endpoints will be specified in the Translational Science Protocol.

2.4 Derivation of endpoints

2.4.1 DLTs

DLTs will be assessed from day 1 of trial treatment up to but not including the time of RC (or 4 weeks after the last dose of atezolizumab if RC does not occur), and will be defined as any of the following events if deemed to be related to the IMP by the SRC:

1. A treatment-related toxicity that delays the RC by more than 2 weeks.
2. Grade 3 or higher haematuria.
3. Any grade 3 or higher immune-mediated toxicity.
4. Any serious adverse event (SAE) considered treatment-related.
5. Grade 4 haematological or ≥ grade 3 non-haematological toxicity considered treatment-related.
6. Any other treatment-related event decided by the SRC to be clinically significant.

If the event is considered by the SRC to be highly unlikely to be related to trial treatment it will not be categorised as a DLT.

To be evaluable for DLTs, participants must have received sufficient treatment as specified in Section 3.2 (dose confirmation DLT evaluable population).

2.4.2 Safety and toxicity

Safety will be reported based on the occurrence of SAEs, SARs and SUSARs. Toxicity will be reported based on AEs and AEoSIs, as graded by NCI CTCAE V5.0 and determined by routine clinical assessments at each centre.

AEs and ARs will be reported from day 1 of trial treatment until 30 days after RC (or planned RC in the event no surgery takes place) or until initiation of new systemic anti-cancer therapy, whichever occurs first. AEoSIs and SAEs will be reported from day 1 of trial treatment until 90 days after RC (or planned RC in the event no surgery takes place) or until initiation of new systemic anti-cancer therapy, whichever occurs first. SAEs will

also be collected following informed consent but prior to initiation of trial treatment, only if the SAE is caused by a protocol-mandated intervention.

SARs and SUSARs will be reported from day 1 of trial treatment until the end of trial. The 'end of the trial' will be defined as the date of the last participant's last data item. This may be either the results of the 2-year data sweep, or the results of the PK/ADA sample analysis, whichever comes last.

2.4.3 Other secondary and exploratory endpoints

- PFS will be calculated from the date of registration to first documented evidence of disease progression (determined according to Response Evaluation Criteria in Solid Tumour (RECIST) 1.1 – see Appendix) or death from any cause, whichever is sooner. Participants who start new anti-cancer treatment prior to progression/death will be censored at the date of starting new treatment. Participants who have not progressed/died at the time of analysis, including those lost to follow-up, will be censored at the last date they were known to be alive and progression-free. Participants who withdraw consent for data collection will be censored at the date of withdrawal if they have not progressed/died by this date.
- Treatment compliance will include details of any dose omissions, dose delays, dose reductions, delays to RC and RC cancellations.
- pCR rate is defined as the proportion of participants categorised as achieving a pCR according to the local pathologist report following RC. Participants who do not undergo RC during the trial and participants without a post-RC pCR assessment will be excluded from the analysis of this endpoint.
- OS will be calculated from the date of registration to death. Participants who are alive at the time of analysis will be censored at the last date they were known to be alive. Participants who withdraw consent for data collection will be censored at the date of withdrawal if they have not died by this date.
- The PK profile may include, but not necessarily be limited to the following PK parameters: maximum plasma concentration (c_{max}); plasma half-life ($t_{1/2}$); time to maximum plasma concentration (t_{max}); area under the concentration time curve (AUC).

2.5 Missing data

Missing data will be chased via a thorough data cleaning process until it is received, confirmed as not available, or the trial is at analysis. Every effort will be made to obtain complete dates for all key data.

Completely missing dates are expected to be very rare and missing dates will be monitored. If a participant is known to have progressed/died but no date for the progression/death is available, their PFS / OS data will be censored at the latest date they were known to be progression-free/alive. If an exact disease progression/death date is unknown, but the month and year is known, the participant will be assumed to have corresponding progression/death on the 15th of that month.

The denominator for non-DLT toxicity summaries for the dose confirmation and dose expansion stages will include all participants included in the corresponding safety populations (defined in Section 3.2 and Section 3.3).

Due to the small number of participants, descriptive summaries and the fact that missing data are not expected, no sensitivity analyses are planned.

3 Populations

3.1 Eligibility

Participants considered eligible for the study are those who fulfil all the inclusion criteria, and none of the exclusion criteria specified in the protocol. All participants in the analysis populations should remain in the trial after registration, unless they explicitly withdraw consent. If a participant is found to be ineligible after registration, they will be included in the analysis population as appropriate, unless written confirmed consent has not been obtained.

3.2 Dose confirmation stages

Dose confirmation DLT evaluable populations

The DLT evaluable population for the dose confirmation stages will be split by treatment route and includes:

- Treatment route 1:
    - For dose confirmation stage 1a (single dose), any participant who receives one dose of atezolizumab via passive instillation, where one dose is defined as at least 80% of the assigned dose.
    - For dose confirmation stage 1b (multiple doses), any participant who receives at least 80% of the total of three times the assigned dose of atezolizumab via passive installation over at least three treatment weeks.
- Treatment route 2:
    - For dose confirmation stage 2a (single dose), any participant who receives one dose of atezolizumab via direct injection, where one dose is defined as at least 80% of the assigned dose.
    - For dose confirmation stage 2b (multiple doses), any participant who receives at least 80% of the total of three times the assigned dose of atezolizumab via direct injection over at least three treatment weeks.

Participants who do not receive these minimum required doses and do not experience a DLT (as described in Section **Error! Reference source not found.**) will not be evaluable for DLTs and will be replaced at the current dose level; these replaced participants will be summarised separately and will be referred to as the DLT non-evaluable population. Assessments of whether each participant is evaluable for DLTs will be performed by the CTRU based on the data provided for each participant on the final day of their treatment, and will be confirmed by the SRC.

Dose confirmation safety populations

The safety population for the dose confirmation stages will be split by treatment route and includes:

- Treatment route 1: all participants who receive at least one dose of atezolizumab via passive instillation during dose confirmation stages 1a (single dose) and 1b (multiple doses).
- Treatment route 2: all participants who receive at least one dose of atezolizumab via direct injection during dose confirmation stages 2a (single dose) and 2b (multiple doses).

3.3 Dose expansion stages

Dose expansion analysis populations

The analysis population for the dose expansion stage will be split by treatment route and includes:

- Treatment route 1: any participant who receives at least one dose of atezolizumab via passive instillation at RD1b during the dose expansion stage. Participants treated with RD1b during the dose confirmation stages will also be included in this analysis population.
- Treatment route 2: any participant who receives at least one dose of atezolizumab via direct injection at RD2b during the dose expansion stage. Participants treated with RD2b during the dose confirmation stages will also be included in this analysis population.

Dose expansion safety populations

The safety population for the dose expansion stage will be split by treatment route and includes:

- Treatment route 1: all participants who receive at least one dose of atezolizumab via passive instillation during the dose expansion stage, including those treated with RD1b during the dose confirmation stages.
- Treatment route 2: all participants who receive at least one dose of atezolizumab via direct injection during the dose expansion stage, including those treated with RD2b during the dose confirmation stages.

4 Data Handling

4.1 Data monitoring

Data will be monitored for quality and completeness by the CTRU. Missing data will be chased until it is received, confirmed as not available, or the trial is at analysis. Any problems with data collection will be discussed at the internal Project Delivery Meetings (PDMs) and, if appropriate, external Trial Management Group (TMG) meetings. All efforts will be made to ensure that as much of the data is present as possible and that reasons are obtained when data is unobtainable.

The CTRU will reserve the right to intermittently conduct source data verification exercises on a sample of participants, which will be conducted by staff from the CTRU. Source data verification will involve direct access to participant notes at the participating hospital sites and the collection of copies of consent forms and other relevant investigation reports. Where source data verification by CTRU is not possible (e.g. due to COVID-19 movement restrictions) alternatives (including virtual/telephone review of source data) may be approved by CTRU and the TMG. A Trial Monitoring Plan will be developed and a Meeting Group Monitoring Schedule including primary endpoint and safety data will be defined and agreed by the TMG if necessary.

The following will also be examined continuously during the course of the trial, as appropriate:

- Accrual
- Data quality and compliance
- Consent
- DLTs
- SAEs/SUSARs
- Withdrawals from the trial
- Protocol deviations/violations.

4.2 SRC

An SRC will be formed for the study. The SRC will consist of the Chief Investigator, Co-Investigators, CTRU statisticians, CTRU trial and data management representatives, at least one Patient and Public Involvement (PPI) member and at least 3 independent members. The independent membership will consist of at least two non-recruiting medics (one of whom will chair the SRC). The SRC will meet at the frequency described in Section 1.6.

The overall aims of the SRC will be to:

- Ensure the safety of participants.
- Review the emerging clinical data and (informally) compare it to expectations from previous experience, contemporary data, and pre-clinical models.

The operation of the SRC will by described in the SRC Terms of Reference document sent out with the protocol.

4.3 Data validation

The Senior Trial Co-ordinator (or their delegate) will perform verification of the forms, as data are received, in accordance with the guidelines developed for the study. This will ensure that data are complete, consistent and up-to-date.

A validation check program will be incorporated into the trial database to verify the data, and discrepancy reports will be generated for resolution by staff at site. Priorities for these validations will be listed in the data management work instructions, and any discrepancies related to participants rights or the safety of participants will be expedited to site for resolution.

Due to the nature of the trial, data management includes 100% data checking. As this study uses remote data entry, checking of data against the case reports forms is not required for data entered electronically.

In addition, the statistician will validate the data to be used in the analysis by performing the following checks:

- Eligibility checks (if not data management validations)
- Sequential dates (if not data management validations)
- Death/progression dates in relation to follow-up dates
- Death/progression dates in relation to registration dates
- Checks for unusual and outlying data (if not data management validations)

- Checks for missing data (are there items of data which are systematically missing/do specific variables have a large amount of missing data etc. – if not data management validations)
- Other checks as deemed appropriate.

SAS will also be used to further validate the data and identify any missing or inconsistent data.

Any inconsistent data will be noted and emailed to the Senior Trial Co-ordinator responsible for the trial (or their delegate). All queries will be resolved if possible, in time for the final report.

4.4 Procedures

The final analysis report will be prepared by the Trial Statistician and reviewed by the Supervising Statistician. The Trial Statistician will manage the report, which may only be accessed by the INVEST trial team. Any other requests for access to the reports should be discussed with the Supervising Statistician and/or CTRU Scientific Lead and, if appropriate, the SRC. The report will contain data on all participants in the relevant population(s) (see Section 3.2 and Section 3.3) that is received and entered onto the trial database by the cut-off date determined for the report. This cut-off date will be discussed and agreed upon at the PDMs to ensure all trial personnel are aware of the date to enable prioritisation, if appropriate, of data entry and data management.

5 Data Analysis

5.1 General calculations

Unless otherwise stated, percentages will be calculated using the total number of participants in the analysis population as the denominator (i.e. including all participants with missing data for that variable).

All percentages and confidence intervals will be rounded to 1 and 2 decimal place(s) respectively. Means, medians, ranges and standard deviations will be summarised to one more decimal place than the data are collected. Values that are below the limit of detection and therefore non-quantifiable will be summarised using the limit of quantification value. For listings, if required, the non-quantifiable value would be reported as an inequality.

All analyses will be conducted using SAS, unless stated otherwise.

5.2 Statistical Considerations

Statistical analysis will be descriptive for all endpoints and will be summarised separately for each treatment route (and dose level, if appropriate).

Participants with protocol deviations and/or eligibility violations with relevant impact upon safety resulting in the participant not being representative of the trial population may be excluded from the relevant analysis populations if deemed appropriate. Participants who do not meet the criteria to be included in each of the analysis sets will have the reasons for omission listed.

5.2.1 Participant replacement

To be evaluable for DLTs, participants in dose confirmation stages 1a and 2a (single dose) must have received one dose of atezolizumab. Participants in dose confirmation stages 1b and 2b (multiple doses) must receive at least three doses of atezolizumab to be considered evaluable for DLTs.

Participants who do not receive the required amount of treatment to be evaluable for DLTs in the dose confirmation stages (one dose in stage 1a and 2a, and at least three doses in stages 1b and 2b) and who do not experience a DLT (as described in Section **Error! Reference source not found.**), will be replaced at the current dose level. This number is expected to be minimal.

Participants in the expansion stages will not be replaced.

5.3 Analysis

5.3.1 Study summary

A CONSORT flow diagram will be used to summarise the course of participants through the study. Protocol

violations will be summarised at the end of the trial, including deviations from the protocol and violations of eligibility criteria upon entry into the trial.

The number of participants who were screened but not registered onto the trial may be summarised if deemed appropriate, using the non-registration log, including reasons for non-entry. The number of participants registered to the study who do not go on to receive any study treatment will also be summarised. The number of withdrawals (clinician or participant withdrawals) of consent to the study will also be summarised, along with reasons for withdrawal. A listing of all withdrawals from the study will be presented, with a participant identifier, the reason for withdrawal, the treatment route, the stage of study (dose confirmation or dose expansion), the dose level and the duration of treatment received before withdrawal.

Median follow-up will be summarised by treatment route for all participants who receive trial treatment, where follow-up is defined as the time from registration to date of death or date last known alive and followed up.

The number of participants who are replaced (see Section 5.2.1) in the dose confirmation stages (the DLT non-evaluable population) will also be summarised, along with the reasons they have been replaced. A listing of all replaced participants will be presented, with a participant identifier, the reason for their replacement, the treatment route, the dose level, and the duration of treatment received before replacement.

Operative and post-operative complications related to RC alone, i.e. not related to trial treatment, may also be summarised by treatment route and dose level, if applicable.

Any confirmed pregnancies reported during the trial will be summarised, including the outcome of pregnancy and outcome of birth.

5.3.2 Baseline characteristics

Baseline characteristics will be tabulated using summary statistics by treatment route and dose level. No statistical testing will be conducted on these data. Baseline characteristics to be summarised include, but may not be limited to:

- Sex
- Ethnicity
- Time from bladder cancer diagnosis to trial registration
- Disease stage
- Type of bladder cancer
- Previous therapies or procedures for bladder cancer
- Tumour profiling
- Eastern Co-operative Oncology Group (ECOG) performance status
- American Society of Anesthesiologists (ASA) physical status classification

5.3.3 Primary endpoint analysis

Dose confirmation stages

The number of participants experiencing DLTs from day 1 of trial treatment up to but not including the time of RC (or 4 weeks after their last dose of atezolizumab if RC does not occur) will be presented, with descriptive summaries of the specific DLTs observed. Summaries will be presented for each treatment route and dose level (if applicable).

This analysis will be based upon the dose confirmation DLT evaluable populations.

Dose expansion stages

The number of SAEs, SARs and SUSARs will be summarised descriptively by treatment route, including the proportion of participants experiencing at least one SAE, the number of SAEs experienced per participant, and the number of SAEs experienced according to seriousness criteria, causality, and body system. Further

breakdown of SAEs by event timing may also be summarised if this is deemed appropriate.

Toxicity will be summarised via AEs and AEoSIs using the NCI CTCAE, Version 5.0. Line listings and the proportion of participants experiencing the maximum grade of each toxicity will be presented by treatment route. Relatedness to either treatment alone, or treatment *and* RC, and further breakdown of AEs and AEoSIs by event timing, may also be summarised if this is deemed appropriate.

This analysis will be based upon the dose expansion safety populations.

5.3.4 Secondary endpoint analysis

<u>All stages</u>

PFS

PFS curves will be calculated using the Kaplan-Meier method. Median PFS estimates and PFS estimates at 3-, 6-, 12- and 24-months post-RC will be presented by treatment route and dose level (if applicable) with corresponding 95% confidence intervals. PFS curves will be presented by treatment route. If appropriate, PFS curves may be presented within each treatment route by the number of doses received (i.e., with participants receiving single doses pooled together, and participants receiving multiple doses pooled together). Participants who start new anti-cancer treatment prior to progression/death will be censored at the date of starting new treatment. The number of participants that this applies to will be summarised. Participants who have not progressed/died at the time of analysis will be censored at the last date they were known to be alive and progression free. Participants who withdraw consent for data collection will be censored at the date of withdrawal if they have not progressed/died by this date. The number of progressions/deaths will also be presented.

This analysis will be based upon the dose confirmation DLT evaluable populations and dose expansion analysis populations.

Treatment compliance

Feasibility of delivering treatment according to protocol schedule will be summarised descriptively overall and by treatment route. This will include the number of participants requiring dose delays and/or reductions, the number of participants withdrawing from treatment, and reasons for dose delays/reductions and withdrawals. Number of doses received (for multiple doses 1b and 2b in the dose confirmation stages, and dose expansion stages only) along with number of doses originally planned, and reasons for stopping treatment will also be presented. The number of omitted doses will be summarised by treatment route, dose level (if applicable) and by treatment week (for multiple doses 1b and 2b in the dose confirmation stages, and dose expansion stages only). For participants allocated to the direct injection route, the volume of the agent injected into the tumour/adjacent bladder wall may be summarised descriptively by dose level.

The number of participants with delays to RC or not undergoing RC, and the reasons for delays to RC/not undergoing RC will be summarised by treatment route and dose level (if applicable).

For patients allocated to the direct injection route, the volume of the agent injected into the tumour/adjacent bladder wall may be summarised descriptively by dose level.

This analysis will be based upon all participants in the dose confirmation safety populations and dose expansion safety populations.

Pathological complete response rate

The proportion of participants experiencing pCR according to the local pathologist report following RC, will be summarised by treatment route and dose level (if applicable). If appropriate, corresponding 95% confidence intervals will also be included.

This analysis will be based upon the dose confirmation DLT evaluable populations and dose expansion analysis populations. Participants who do not undergo RC during trial will be excluded from this analysis, and the reasons for not undergoing RC will be summarised within the treatment compliance analysis results. Participants who undergo RC but are missing a post-RC pCR assessment will also be excluded from this analysis, and corresponding reasons for missing post-RC pCR assessments will be summarised.

Dose confirmation stages

The number of SAEs, SARs and SUSARs will be summarised descriptively by treatment route and dose level (if applicable), including the proportion of participants experiencing at least one SAE, the number of SAEs experienced per participant, and the number of SAEs experienced according to seriousness criteria, causality, and body system. Further breakdown of SAEs by event timing may also be summarised if this is deemed appropriate.

Toxicity will be summarised via AEs and AEoSIs using the NCI CTCAE, Version 5.0. Line listings and the proportion of participants experiencing the maximum grade of each toxicity will be presented by treatment route and dose level (if applicable). Relatedness to either treatment alone, or treatment *and* RC, and further breakdown of AEs and AEoSIs by event timing, may also be summarised if this is deemed appropriate.

This analysis will be based upon the dose confirmation safety populations.

5.3.5 Exploratory endpoint analysis

OS

OS curves will be calculated using the Kaplan-Meier method. Median OS estimates and OS estimates at 6-, 12- and 24-months post-RC will be presented by treatment route and dose level (if applicable) with corresponding 95% confidence intervals. If deemed appropriate, OS curves may be presented within each treatment route by the number of doses received (i.e., with participants receiving single doses pooled together, and participants receiving multiple doses pooled together). Participants who have not died at the time of analysis will be censored at the last date they were known to be alive. Participants who withdraw consent for data collection will be censored at the date of withdrawal if they have not died by this date.

The number of participants who have died and causes of death will also be presented.

This analysis will be based upon the dose confirmation DLT evaluable populations and dose expansion analysis populations.

Serum and urinary PK

Concentration-time data will be plotted for each participant at the time points given in Table 1 and Table 2. PK parameters to be estimated may include, but not necessarily be limited to c_{max}, $t_{1/2}$, t_{max} and AUC.

This analysis will be based upon the dose confirmation DLT evaluable populations and dose expansion analysis populations.

Table 1 - Serum and urine collection schedule for PK (single dose trial treatment only)

**Assessment time point**	**Day 1 Week 1**								**Post-treatment, pre-RC**
Scheduling window	-2-0 hours pre-dose	0.5 hours (+/- 10 mins) post-dose	0-2 hours post-dose	1-hour (+/- 10 mins) post-dose	2-4 hours post-dose	3 hours (+/- 10 mins) post-dose	4-6 hours post-dose	6 hours (+/- 30 mins) post-dose	1 week post treatment -3/+7 days
**Serum for PK**	X	X		X		X		X	X
**Urine for PK**	X		X		X		X		X

Table 2 - Serum and urine collection schedule for PK and ADA (multiple dose trial treatment only)

**Assessment**	**Day 1**	**Day 1 Week 1**	**Day 1**	**Day 1**	**Post-**

time point	Week 1-6								Week 2-6	Week 3 & 6	treatment, pre-RC
Scheduling window	-2-0 hours pre-dose	0.5 hours (+/- 10 mins) post-dose	0-2 hours post-dose	1-hour (+/- 10 mins) post-dose	2-4 hours post-dose	3 hours (+/- 10 mins) post-dose	4-6 hours post-dose	6 hours (+/- 30 mins) post-dose	0-2 hours post-dose	0-2 hours pre-dose	1 week post treatment – 3/+7 days
**Serum for PK**	X	X		X		X		X	X		X
**Urine for PK**	X		X		X		X		X		X
**Serum for ADAs**	X^									X*	X

^Week 1 of treatment only.

**If participant is not scheduled to receive 6 weeks of trial treatment, the serum sample for ADA can be taken on the final scheduled trial treatment visit instead of on week 6.*

ADAs

The numbers and proportions of ADA-positive participants and ADA-negative participants, at baseline (baseline prevalence) and after start of trial treatment (post-baseline incidence) will be summarised by treatment route and, if applicable, dose level. Post-baseline incidence may also be summarised by timepoint, if applicable, as given in Table 2.

When determining post-baseline incidence, participants are considered to be:

- ADA positive if they are ADA negative or have missing data at baseline, but develop an ADA response following start of trial treatment (treatment-induced ADA response), or if they are ADA positive at baseline and the titre of one or more post-baseline samples is at least 0.60 titre unit greater than the titre of the baseline sample (treatment-enhanced ADA response).

- ADA negative if they are ADA negative or have missing data at baseline and all post-baseline samples are negative, or if they are ADA positive at baseline, but do not have any post-baseline samples with a titre that is at least 0.60 titre unit greater than the titre of the baseline sample (treatment unaffected).

This analysis will be based upon the dose confirmation DLT evaluable populations and dose expansion analysis populations, but for those participants with multiple doses only. Participants missing a baseline ADA assessment will be classed as missing in the baseline prevalence calculation. Participants who do not have at least one post-baseline atezolizumab ADA assessment will be excluded from the post-baseline ADA incidence summaries.

6 Reporting and Dissemination of the Results

Once the final analysis has been completed the results will be written up into a statistical report and presented to the TMG who will discuss them and decide if any further analysis or investigation is required. After this, members of the TMG will write up the results with a view to submitting a publication piece to a peer-reviewed scientific journal. All publications (abstracts and full manuscripts) must be reviewed by the Chief Investigator (CI) or Co-CI, each member of the TMG and pharmaceutical companies if contractually obliged, prior to submission. Interim safety data from the dose confirmations stages may be submitted in abstract form to relevant conferences, as required and in discussion with the TMG and SRC.

7 References

1. Chavan, S., et al., *International variations in bladder cancer incidence and mortality.* Eur Urol, 2014. **66**(1): p. 59-73.
2. Svatek, R.S., et al., *The economics of bladder cancer: costs and considerations of caring for this disease.* Eur Urol, 2014. **66**(2): p. 253-62.
3. Babjuk, M., et al., *EAU Guidelines on Non-Muscle-invasive Urothelial Carcinoma of the Bladder: Update 2016.* Eur Urol, 2017. **71**(3): p. 447-461.
4. Babjuk, M., et al., *European association of urology guidelines on non-muscle-invasive bladder cancer (TaT1 and carcinoma in situ)-2019 update.* 2019. **76**(5): p. 639-657.
5. Gontero, P., et al., *Prognostic factors and risk groups in T1G3 non-muscle-invasive bladder cancer patients initially treated with Bacillus Calmette-Guerin: results of a retrospective multicenter study of 2451 patients.* Eur Urol, 2015. **67**(1): p. 74-82.
6. Sylvester, R.J., et al., *Predicting recurrence and progression in individual patients with stage Ta T1 bladder cancer using EORTC risk tables: a combined analysis of 2596 patients from seven EORTC trials.* Eur Urol, 2006. **49**(3): p. 466-5; discussion 475-7.
7. Mostafid, A.H., et al., *Therapeutic options in high-risk non-muscle-invasive bladder cancer during the current worldwide shortage of bacille Calmette-Guerin.* Eur Urol, 2015. **67**(3): p. 359-60.
8. van Hooren, L., et al., *Local checkpoint inhibition of CTLA-4 as a monotherapy or in combination with anti-PD1 prevents the growth of murine bladder cancer.* Eur J Immunol, 2017. **47**(2): p. 385-393.
9. Balar, A.V., et al., *Atezolizumab as first-line treatment in cisplatin-ineligible patients with locally advanced and metastatic urothelial carcinoma: a single-arm, multicentre, phase 2 trial.* 2017. **389**(10064): p. 67-76.
10. Rosenberg, J.E., et al., *Atezolizumab in patients with locally advanced and metastatic urothelial carcinoma who have progressed following treatment with platinum-based chemotherapy: a single-arm, multicentre, phase 2 trial.* 2016. **387**(10031): p. 1909-1920.
11. Powles, T., et al., *Atezolizumab versus chemotherapy in patients with platinum-treated locally advanced or metastatic urothelial carcinoma (IMvigor211): a multicentre, open-label, phase 3 randomised controlled trial.* 2018. **391**(10122): p. 748-757.
12. Roche, *Internal Communication*. 2021.
13. Woodcock, V.K., et al., *A phase I study to assess the safety and tolerability of intravesical pembrolizumab in recurrent non-muscle invasive bladder cancer (NMIBC).* 2019. **37**(7_suppl): p. 406-406.
14. Moe, A., et al., *A phase I open label dose-escalation study to evaluate the tolerability, safety and immunological efficacy of sub-urothelial durvalumab injection in adults with muscle-invasive or high-risk non-muscle-invasive bladder cancer (SUBDUE-1, SUB-urothelial DUrvalumab injection-1 study): clinical trial protocol.* 2021.
15. Hellenic GenitoUrinary Cancer Group. *Intravesical Administration of Durvalumab (MEDI4736) to Patients With High-risk, Non-muscle-invasive Bladder Cancer (NMIBC). A Phase II Study With Correlative*. 2018 04/11/2020 [cited 2021 19/10/2021]; Available from: https://clinicaltrials.gov/ct2/show/study/NCT03759496.

16. Meghani, K., et al., *First-in-human Intravesical Delivery of Pembrolizumab Identifies Immune Activation in Bladder Cancer Unresponsive to Bacillus Calmette-Guérin.* 2022.
17. Davis, N., et al., *Incidence, cost, complications and clinical outcomes of iatrogenic urethral catheterization injuries: a prospective multi-institutional study.* 2016. **196**(5): p. 1473-1477.
18. Feneley, R.C., et al., *Urinary catheters: history, current status, adverse events and research agenda.* 2015. **39**(8): p. 459-470.
19. Mangera, A., et al., *Contemporary management of lower urinary tract disease with botulinum toxin A: a systematic review of botox (onabotulinumtoxinA) and dysport (abobotulinumtoxinA).* 2011. **60**(4): p. 784-795.
20. Harrington, K., et al., *Guidelines for preclinical and early phase clinical assessment of novel radiosensitisers.* 2011. **105**(5): p. 628-639.

8 Appendices

8.1 Appendix 1 – Trial flow diagram

Figure 1 - Trial flow diagram

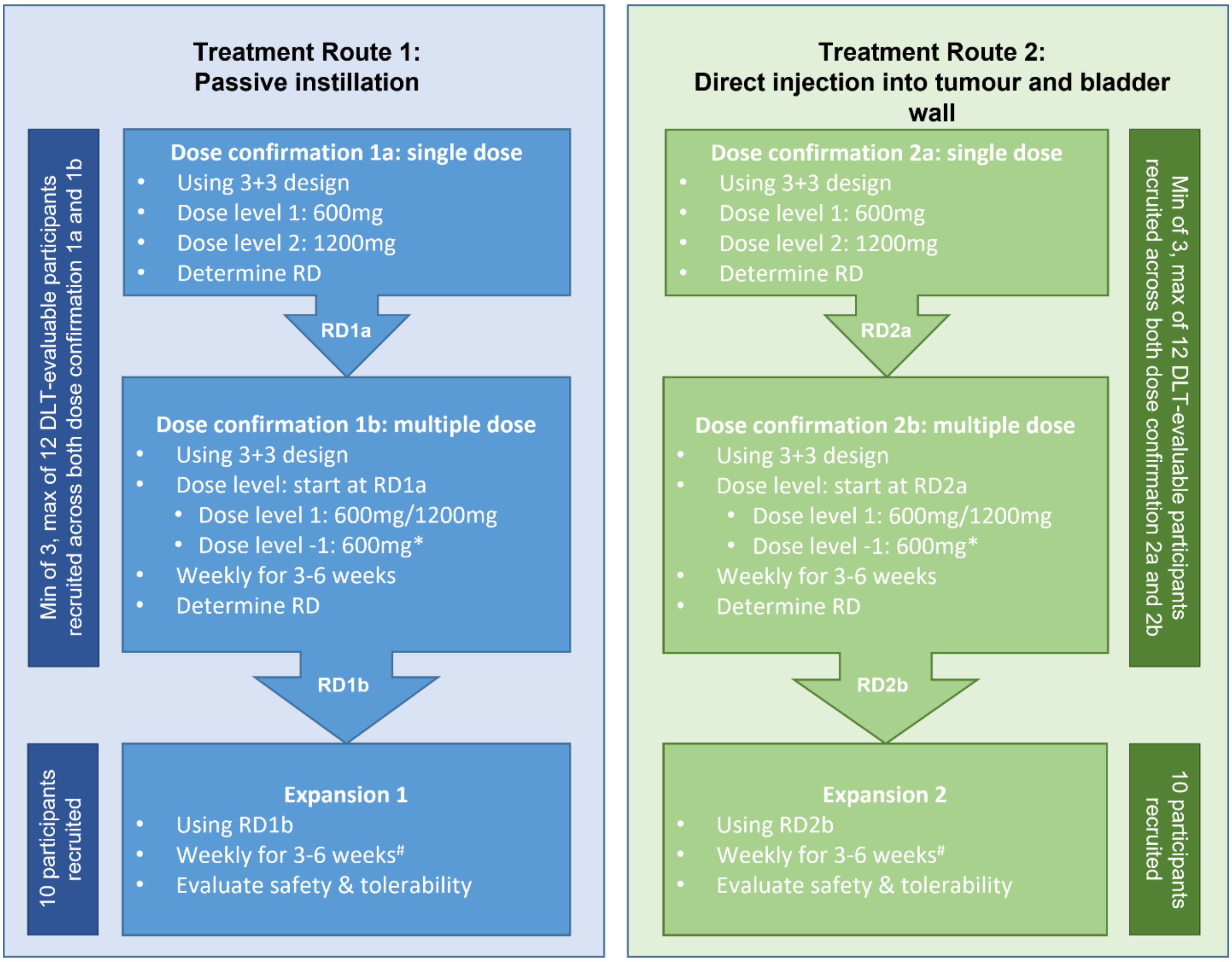

RD = Recommended dose
The first single dose and multiple dose participants in each treatment route will be recruited and observed for the DLT reporting period, prior to any further participants being recruited to that respective first cohort.

* Only if RD1a / RD2a is 1200mg
# Single dose (RD1a / RD2a) if multiple doses are not tolerated in dose confirmation 1b / 2b respectively.

8.2 Appendix 2 – DLT schema for both treatment routes

Figure 2 - DLT schema for both treatment routes

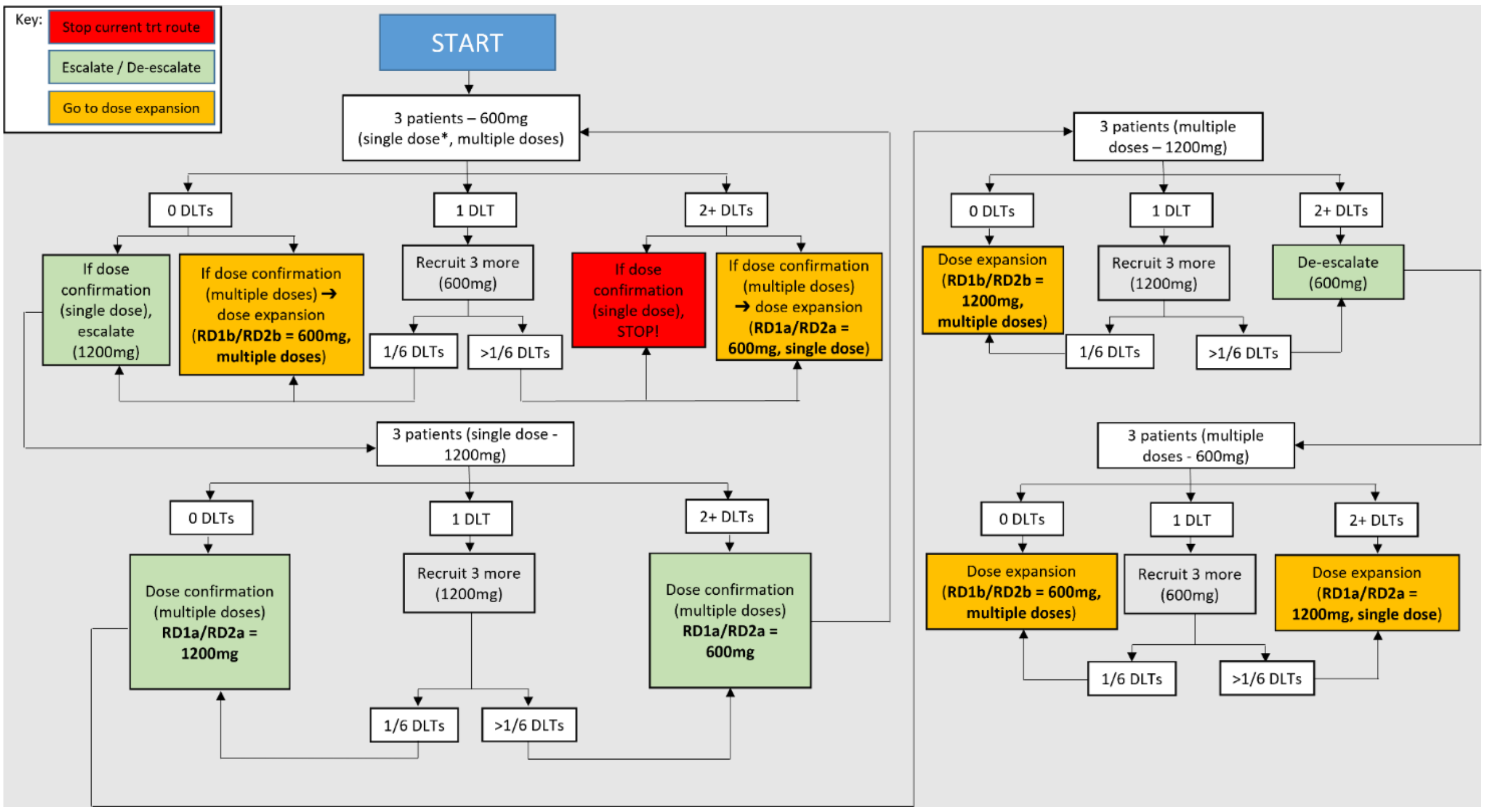

* Begin with dose confirmation 1a/2a – single dose. Dose confirmation 1b/2b for multiple doses follows (see Section 7.3).
The first single dose and multiple dose participants in each treatment route will be recruited and observed for the DLT reporting period, prior to any further participants being recruited to that respective first cohort.

8.3 Appendix 3 – RECIST 1.1 guideline

RECIST 1.1 guideline (published January 2009) can be obtained at: https://recist.eortc.org/recist-1-1-2/

8.4 Appendix 4 - Approval of Analysis Plan

Clinical Trials Research Unit (CTRU)

The following final analysis plan, V2.0 June 2024, for the INVEST study, has been approved by the following personnel. Any signed amendments to the plan will be filed with this document.

Trial Statistician: Ethan Senior

Print Name: E. Senior

Date: 10/06/2024

Supervising Statistician: Jessica Kendall

Print Name:

Date:

CTRU Scientific Lead: Fiona Collinson

Print Name:

Date:

Trial Management Lead: Jamie Oughton

Print Name:

Date:

Senior Trial Co-ordinator: Ruby Smith Whelan

Print Name: 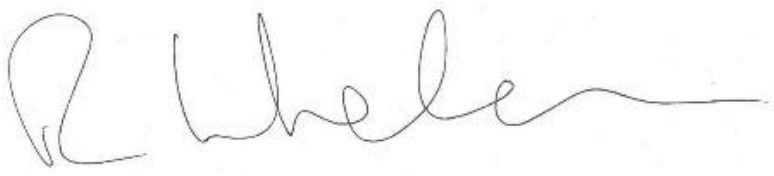

Date: 02/10/2024

Chief Investigator: Syed Hussain

Print Name:

Date:

Additional information:

Sign-off for Jessica Kendall dated 11/06/2024 can be found here: P:\CTRU\Projects\Cancer\Early phase\INVEST\Stats\02_SAP\SAP_V2.0_240610\Sign-Off V2.0\RE INVEST Statistical Analysis Plan June 2024 V2.0 Sign-off_JK.msg

Sign-off for Fiona Collinson dated 11/09/2024 can be found here: P:\CTRU\Projects\Cancer\Early phase\INVEST\Stats\02_SAP\SAP_V2.0_240610\Sign-Off V2.0\RE INVEST Statistical Analysis Plan June 2024 V2.0 Sign-off_FC.pdf

Sign-off for Jamie Oughton dated 11/06/2024 can be found here: P:\CTRU\Projects\Cancer\Early

phase\INVEST\Stats\02_SAP\SAP_V2.0_240610\Sign-Off V2.0\RE INVEST Statistical Analysis Plan June 2024 V2.0 Sign-off_JO.msg

Sign-off for Syed Hussain dated 29/07/2024 can be found here: P:\CTRU\Projects\Cancer\Early phase\INVEST\Stats\02_SAP\SAP_V2.0_240610\Sign-Off V2.0\INVEST Final Statistical Analysis Plan v2.0 June 2024 signed SH.pdf
