# Supplementary material for: INtraVESical immunoTherapy (INVEST) prior to radical cystectomy for bladder cancer: trial protocol
Source: BJU Int. 2026 Jul 27;138(4):607–19. doi: 10.1111/bju.70387 (PMC13550807; doi:10.1111/bju.70387)
Supplement: Supplementary file 2 [file BJU-138-607-s003.pdf]

Delete this line, then print first page of Information Sheet and Consent Form on Trust/Hospital headed paper

INVEST

Phase I study of Intravesical immunotherapy for bladder cancer patients undergoing radical cystectomy

PARTICIPANT INFORMATION SHEET

A large-print and/or electronic version of this sheet is available on request.

You have been invited to take part in a research study called "INVEST". Before you decide if you want to take part, we would like to explain why we are doing the research, how we will use the information we have about you, and what the study will involve.

Please read this information carefully and discuss it with others if you like. Ask us if anything is unclear, or if you would like more information.

Once you have read this information, your doctor will talk to you about the study again and you can ask any questions you like.

- **Part 1** tells you the purpose of this study and what will happen to you if you take part.
- **Part 2** gives you more detailed information about the conduct of the study.
- **Part 3** explains how your information will be used if you agree to take part. An optional extra section goes into more detail about this. You don't need to read the optional section if you feel the 'quick access guide' told you what you wanted to know.

Take time to decide whether you wish to take part.

How to contact us

If you have any questions about this study, please talk to your study doctor/nurse at

<<Enter PI, nurse name >>

<< Contact details for site>>

Thank you for reading this information sheet.

Part 1 - Overview

Background information

Bladder cancer is a common disease that can be difficult to manage. In some people, their cancer can be treated using medicines given into the bladder, whilst others need removal of their entire bladder. Unfortunately, most patients develop multiple bladder tumours, and a minority develop progression to muscle-invasion of the tumours, spread of the cancer to other areas of the body, and death. We are investigating new treatments that could be used to control bladder cancer and reduce the need for bladder removal in the future.

What is the purpose of the study?

In this study, known as INVEST, we are investigating a drug called atezolizumab. This is an immunotherapy treatment (a type of cancer treatment that helps your immune system fight cancer) that is usually given by an intravenous method (into the blood stream via a vein). We know that this drug works on more advanced bladder cancer when given by this method. We think this drug may be more effective when given directly into the bladder. We want to explore if we can give this drug safely into the bladder, and also whether there is any indication that it may work on the cancer and the immune system when given this way. To determine this, we will give the drug to a small number of participants who are planned to have radical cystectomy (bladder removal surgery) as a treatment for their bladder cancer. This means we can check for any side effects participants may experience, and we can investigate how the drug works on the cancer. "Study treatment" within this information sheet refers to treatment with atezolizumab.

Why have I been chosen?

You have been chosen as you have been diagnosed with bladder cancer and are planned to have bladder removal surgery in the near future.

Do I have to take part?

No, your participation in INVEST is voluntary. If you decide to take part, you will be asked to sign a consent form, but you are still free to withdraw at any time and without giving a reason. Your treatment and care (for your bladder cancer and afterwards) will not be affected in any way. Your bladder removal surgery will take place as planned whether you take part in the study or not.

If I want to, will I definitely be able to take part?

Unfortunately, no. Although your doctor thinks you might be suitable to take part as you will have already had a biopsy confirming that you have bladder cancer, they will still need to carry out some tests and ask you some questions to make sure you are suitable. These are known as **"eligibility screening tests"**. For this study, these include:

- An assessment of your medical history
- A physical examination
- Blood and urine tests

- Pregnancy test (if you are a woman of childbearing potential)
- A computerised tomography (CT) scan of your chest, abdomen, and pelvis
- An electrocardiogram (ECG)

You would need to have most of these tests done if you were not going to take part in this study, and some of them may already have been done as part of the investigations of your cancer.

If the eligibility screening tests show that it is not appropriate for you to take part, your doctor will discuss your alternative standard treatment options with you.

<u>What is the standard treatment?</u>

Standard treatment for your bladder cancer is surgery alone to remove your bladder (radical cystectomy). You will have surgery as planned even if you do not enter the study.

<u>What is the new treatment that is being tested?</u>

We are investigating whether we can give atezolizumab safely into the bladder directly, and whether there is any suggestion that it may work on the cancer and the immune system. For further details on atezolizumab, please refer to the sections titled **"What is the purpose of the study?"** and **"Possible risks associated with atezolizumab".**

This study aims to explore giving atezolizumab in two new methods into the bladder. Firstly, by introducing it directly into the bladder (passive instillation). Secondly, by injecting it directly into the tumour within the bladder (direct injection).

Passive instillation

This involves filling the bladder with a solution of the atezolizumab using a catheter (a tube that goes into your bladder through your urethra, the tube which carries urine out of the body), leaving it in place for approximately an hour, and then draining it. This is done under local anaesthetic. This way of giving drugs is regularly used in standard care for other types of earlier bladder cancer using drugs such as BCG.

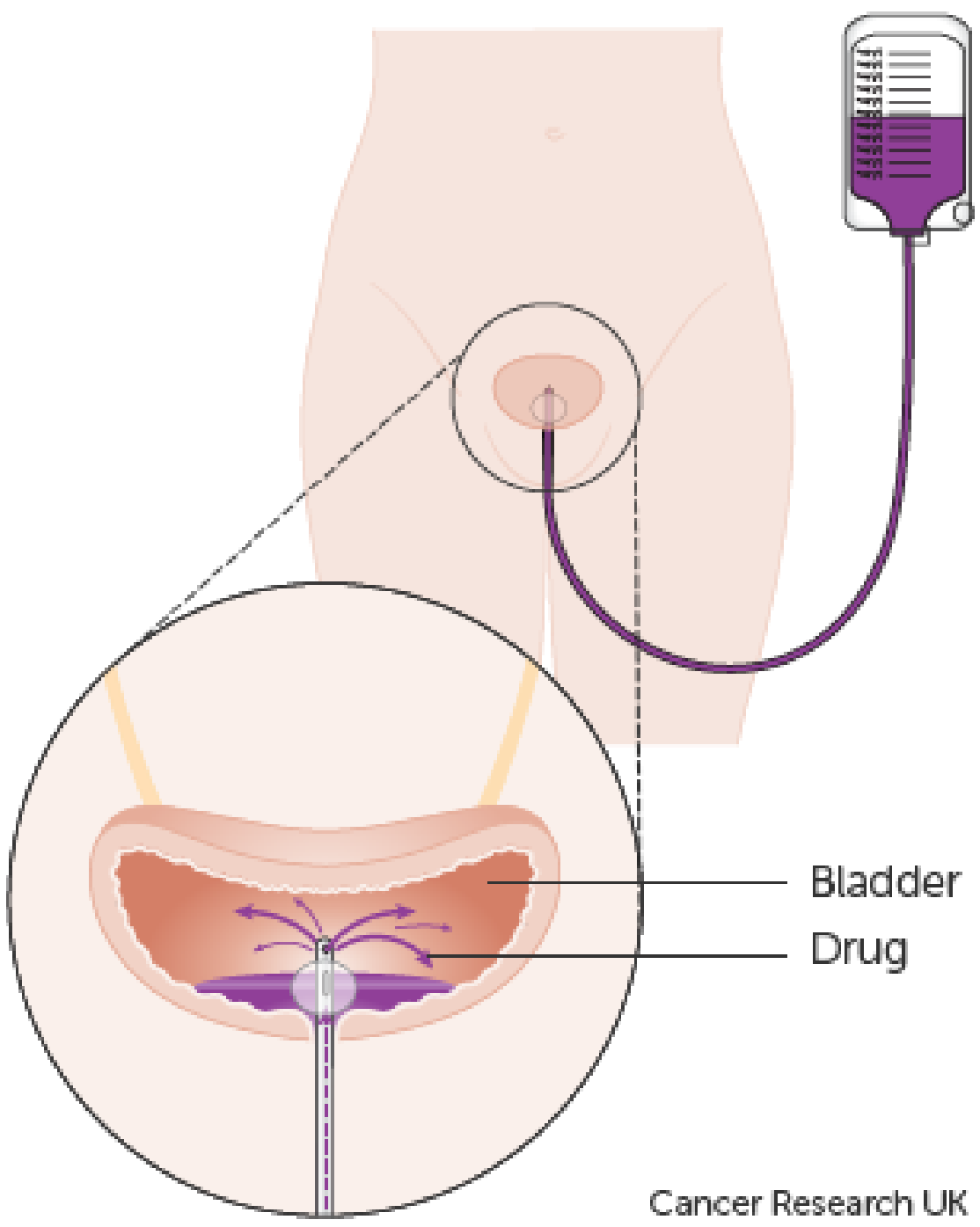

1 https://www.cancerresearchuk.org/about-cancer/bladder-cancer/treatment/early/bcg

Direct injection

This involves injecting the atezolizumab directly into the tumour within the bladder. A

narrow telescope (called a cystoscope) is passed through the urethra into the bladder. The drug is then injected into the tumours and the bladder wall directly. This is done under local anaesthetic. This method is used regularly for Botox injections into the bladder to treat incontinence.

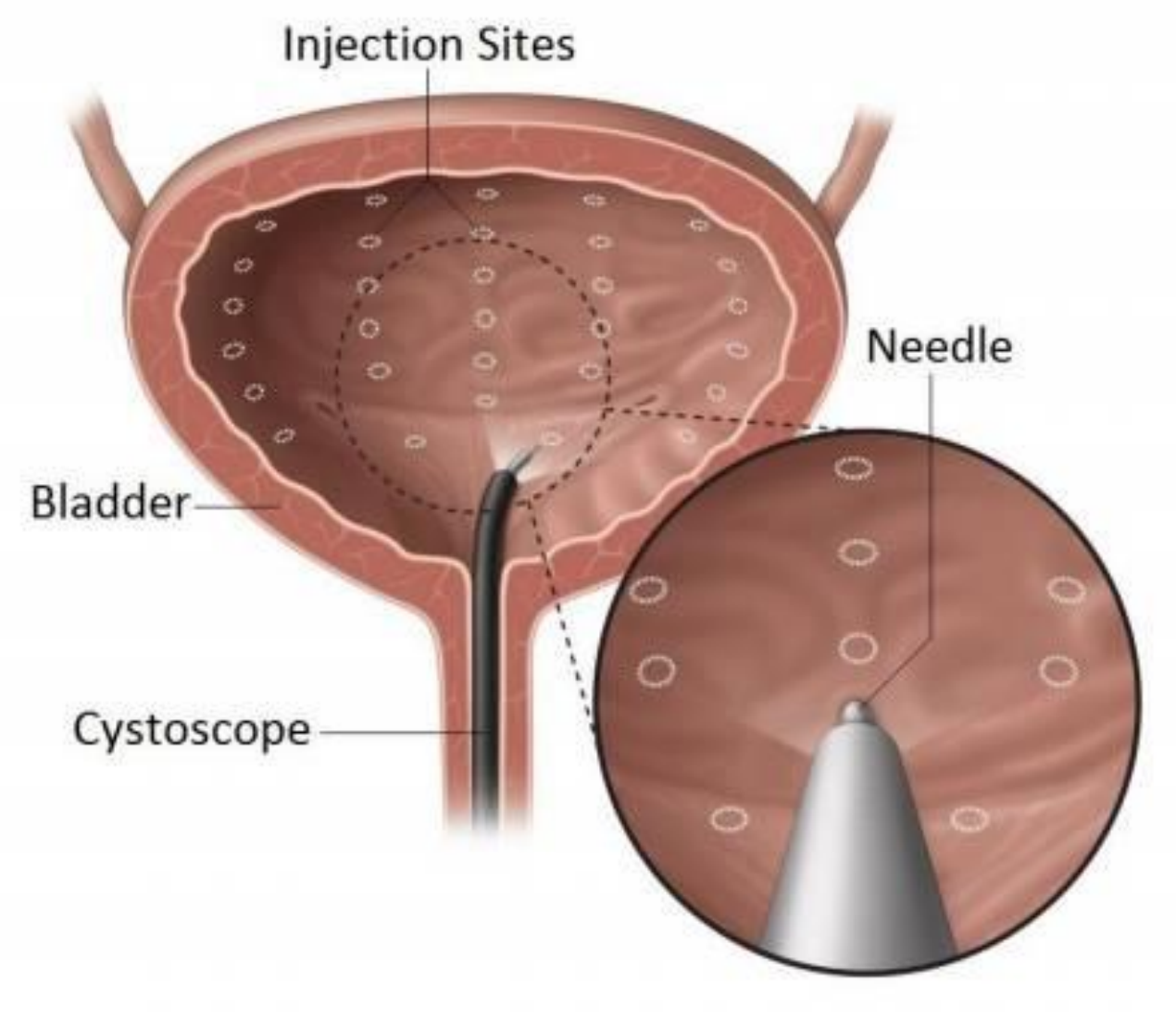

2 https://www.mkuh.nhs.uk/patient-information-leaflet/botox-injections-for-the-treatment-of-an-overactive-bladder-2

As these approaches are both new ways of giving atezolizumab the study is also exploring what dose to use, and how many times it can be used. The study is investigating giving both a single dose, or multiple doses (weekly over 3-6 weeks) and using doses of 600mg or 1200mg.

All participants will receive atezolizumab. There is no placebo/dummy drug used in this study.

How is study treatment allocated?

Upon entry into the study, you will be told whether you will be receiving the drug by passive instillation or direct injection. This will be decided by the study management team, based on logistics at the hospital, and which treatment route has a place available. You are not able to choose which treatment route you receive.

You will also be told the dose you will receive (either 600mg or 1200mg) and whether this will be for a single dose (a one off), or multiple doses (once per week for 3-6 weeks). This will be determined by the study management team based on how many participants have been treated so far in the study and any side effects seen.

Please see the table on the next page for a full overview of the study treatment groups for the treatment routes, doses and dose schedules. You will only ever be allocated to one of these groups, consisting of one treatment route, one dose level and one dose schedule.

Table explaining the treatment routes, doses and dose schedules for passive instillation

Treatment route	Stage	Dose level	Dose schedule	Details
**Passive instillation**	**Dose confirmation 1a**	600mg	Single dose (1 week)	This is the starting dose for the first group of 3 participants
		1200mg	Single dose (1 week)	If minimal side effects are seen at 600mg for the first 3 participants, we will increase the dose to 1200mg for the next group of 3 participants, or a further group of 3 participants will receive 600mg
	**Dose confirmation 1b**	600mg	Multiple dose (once weekly for 3-6 weeks)	From all the groups in dose confirmation stage 1a, if 600mg but not 1200mg was seen to be safe (side effects seen at 1200mg), we will investigate multiple doses of 600mg for a group of 3 participants
		1200mg	Multiple dose (once weekly for 3-6 weeks)	From all the groups in dose confirmation stage 1a, if 1200mg was seen to be safe (minimal side effects), we will investigate multiple doses of 1200mg with a group of 3 participants, and reduce to multiple doses of 600mg for a further group of 3 participants if side effects are seen in the 1200mg group
	**Dose expansion 1**	600mg or	Single dose or multiple dose	Depending on the results of dose confirmation 1a and 1b, we will use either single or multiple doses of 600mg or 1200mg for a group of 10 participants
		1200mg		

<u>Table explaining the treatment routes, doses and dose schedules for direct injection</u>

Treatment route	Stage	Dose level	Dose schedule	Details
**Direct injection**	**Dose confirmation 2a**	600mg	Single dose (1 week)	This is the starting dose for the first group of 3 participants
		1200mg	Single dose (1 week)	If minimal side effects are seen at 600mg for the first 3 participants, we will increase the dose to 1200mg for the next group of 3 participants, or a further group of 3 participants will receive 600mg
	**Dose confirmation 2b**	600mg	Multiple dose (once weekly for 3-6 weeks)	From all the groups in dose confirmation stage 2a, if 600mg was seen to be safe but not 1200mg (side effects seen at 1200mg), we will investigate multiple doses of 600mg for a group of 3 participants
		1200mg	Multiple dose (once weekly for 3-6 weeks)	From all the groups in dose confirmation stage 2a, if 1200mg was seen to be safe (minimal side effects), we will investigate multiple doses of 1200mg with a group of 3 participants, and reduce to multiple doses of 600mg for a further group of 3 participants if side effects are seen in the 1200mg group
	**Dose expansion 2**	600mg or<br>1200mg	Single dose or multiple dose	Depending on the results of dose confirmation 2a and 2b, we will use either single or multiple doses of 600mg or 1200mg for a group of 10 participants

How long does study treatment go on?

All study treatment will take place between your diagnosis of bladder cancer and your bladder removal surgery. Study treatment will not delay your surgery.

If you are allocated to receive a single dose of the drug, study treatment will take place 1-8 weeks before surgery. If you are allocated to receive multiple doses of the drug, you will receive study treatment for 3-6 weeks before surgery, with your surgery taking place 1-8 weeks after your last dose of study treatment. The study treatments will be fitted in before your planned surgery and your surgery will not be delayed by going into the study.

What if the study treatment doesn't help?

This study is the first time that this drug has been given by these methods (directly into the bladder via passive instillation or direct injection). Our primary aim of the study is to check the safety of these methods of giving the drug and how tolerable it is for participants. We will not find out definitively in this study whether the study treatment will improve cancer treatment, but we will look at any response that we see in the bladder tumour when it is removed. Standard treatment (bladder removal surgery) will still be planned, regardless of the study treatment.

What will happen to me if I take part?

If you do take part in this study, you will be required to attend all visits that are made for you. For a table showing the study visit schedule, please see **Appendix 1 – Visit schedule**.

There are some procedures that will happen at all your visits. Your doctor will check that you have been well while on the study treatment, will check your vital signs (blood pressure, pulse rate, respiratory rate and temperature) and you may have a physical examination and an ECG if your doctor thinks this is medically necessary. Your doctor will review with you what medications you have been taking and ask if you have experienced any health problems such as side-effects since your last visit.

There are three different kinds of blood and urine samples taken as part of this trial. The first are for blood and urine tests, to confirm you are well enough to take part/continue taking part. These are taken regularly throughout the trial. The second are blood and urine samples which will be used to analyse pharmacokinetics (PK) and anti-drug antibodies (ADA) (see section titled **"Blood and urine samples for PK/ADA"** for details). These are taken just before, during and shortly after completing study treatment. The final type are blood and urine samples taken and stored and used for future translational work. These are taken before and after study treatment. Please see the section titled **"Additional biological samples"** for more detail about these samples.

Registration

Once you have had any questions answered and have completed the consent form with

your doctor, arrangements will be made for you to start the study. Firstly, we will collect some demographic information about you (date of birth, ethnicity, sex, NHS number). We will also confirm your diagnosis of bladder cancer, and your expected date of bladder removal surgery.

We will then register you as a potential participant and allocate your study treatment – please see the section titled **"How is study treatment allocated?"** for details of how this works.

A sample of tissue from the biopsy which confirmed your diagnosis of bladder cancer will be taken for use in future translational research. No extra biopsy is needed. Please see the section below titled **"Additional biological samples"** for further details.

Eligibility

Once you have been registered, you will need to have all the eligibility screening tests done listed in the section above (**"If I want to, will I definitely be able to take part?"**) if you have not already had them done.

The study has limited places available. The way the study works also means that the study can be paused at various times, to look at the information from those already taking part. For these reasons it cannot be guaranteed that you will be able to take part, even if you have given consent and had the eligibility screening tests. This will be rare, and we will try to minimise this happening.

Once you are confirmed as eligible, your study treatment visits will be booked in.

Optional magnetic resonance imaging-positron emission tomography (MRI- PET)

At registration, you will be asked if you are willing to have an optional magnetic resonance imaging-positron emission tomography (MRI- PET) scan of your bladder. This will be taken at the following times:

- after you have been confirmed as eligible to take part but before study treatment, and;
- following the end of study treatment but prior to bladder removal surgery.

Please see **appendix 2** for the full details of the MRI-PET sub-study before you decide whether you would like to take part.

Study treatment visits

Prior to starting study treatment

If it is longer than three days since your eligibility blood and urine tests were done, blood and urine tests to confirm you are still well enough to start the study treatment will be taken before study treatment starts.

These tests may be taken up to 3 days prior to receiving your first dose of study treatment.

On the day of study treatment

On the day of receiving each dose of atezolizumab (study treatment), you will have blood and urine samples for PK/ADA analysis collected.

Blood samples will be collected at the following times:

- within 2 hours prior to receiving each dose of study treatment,
- at intervals of 0.5, 1, 3, and 6 hours following your first study treatment, and;
- between 0-2 hours after receiving study treatment on weeks 2-6 of study treatment (if you are allocated to receive multiple doses).

Urine samples will be collected at the following times:

- within 2 hours prior to receiving each dose of study treatment,
- at intervals of 0-2, 2-4 and 4-6 hours following your first study treatment, and;
- between 0-2 hours after receiving study treatment on weeks 2-6 of study treatment (if you are allocated to receive multiple doses).

Samples of blood and urine for future translational research will also be taken prior to each dose of study treatment. Please see the section below titled **"Additional biological samples"** for further details.

For potential side effects of the study treatment, please see the section below titled **"might the study treatment have any unwanted effects?".**

Prior to continuing study treatment

Those who are allocated to receive a single dose will now move on to follow-up visits. For participants who are allocated to have multiple doses of study treatment, blood and urine tests will be taken prior to each subsequent week of treatment, to confirm you are still well enough to continue study treatment.

You will also have a pregnancy test every other week while you are on study treatment, if you are if you are a woman of childbearing potential.

The blood and urine tests and pregnancy test may be taken up to 3 days prior to receiving each dose of study treatment.

Follow-up visits

Most of the data we will collect will be collected prior to your bladder removal surgery. We will also collect data at the time of your surgery. There will also be visits following surgery. Most of these would have taken place as standard of care, for example your stay in hospital following surgery, and regular follow-ups to monitor your health after surgery. Additional blood tests that are specific to the trial will be taken to monitor your condition, as well as blood, urine and tissue samples. Details of the follow-up visits are included below.

After finishing study treatment, before bladder removal surgery

Approximately 1 week following your final dose of study treatment, you will have the following assessments.

Samples of blood and urine for PK/ADA analysis will be collected 1 week following the end of all study treatment.

Samples of blood and urine for future translational research will also be taken 1 week following the end of all study treatment. Please see the section below titled **"Additional biological samples"** for further details.

You will have a CT scan of your chest, abdomen and pelvis. This is additional to scans you would have as standard of care.

If you agreed to take part in the MRI-PET sub-study, you will have your final scan at this time.

At bladder removal surgery

Your surgery will take place within 1-8 weeks after the end of study treatment. Blood and urine tests will be taken to confirm you are still well enough to undergo surgery. In addition, a tissue sample for future translational research will be taken from your bladder once it has been removed. Please see the section below titled **"Additional biological samples"** for further details.

Following bladder removal surgery

Following surgery, you will attend regular follow-up visits (approximately once a month for three months) to monitor your recovery and record any complications you are experiencing. At these visits, you will have blood tests to monitor your response to the study treatment.

Samples of blood and urine for future translational research will also be taken 4 or 8 weeks following your surgery. Please see the section below titled **"Additional biological samples"** for further details.

Approximately a month following surgery, you will have an intravenous urogram (IVU).

You will have a final CT scan of your chest, abdomen and pelvis at 12 weeks following surgery. These scans would all take place as part of standard of care following surgery.

Follow up visits will continue until approximately 3 months after surgery, at which point you will have no further visits.

2-year follow-up

In addition, we will collect data on your overall health status at the time the study finishes and the status of your cancer. We will also check whether you have experienced any side effects and collect information on these for as long as the study is open, by the trial team at your site checking your records. This will not require you to come in for a study visit.

<u>Might the study treatment have any unwanted effects?</u>

Possible risks associated with atezolizumab

As with all types of cancer treatment, you may experience some side effects. We believe it is safe to give this drug into the bladder, but as this is the first time this has been done, we cannot be sure. Other doctors have given other immunotherapy drugs (pembrolizumab and durvalumab) into the bladder, and they report these drugs were well tolerated without untoward side effects.

Small amounts of the drug will be absorbed into the blood stream and there is a small risk that this could cause some other immune side effects which can be seen when the drug is given directly into a vein (the usual way of using this drug). Atezolizumab and other immune drugs have been used for many years now via direct blood injection, and doctors understand how to monitor and treat these immune side effects. If unexpected serious side effects are seen, we will stop the atezolizumab treatment.

To help us protect your safety and the safety of other participants like you, you should always tell your doctor about any health events you have experienced during your time on the study or afterwards (such as having to go to hospital for any reason).

Giving anti-cancer drugs into the bladder does not normally have as many other side-effects as having tablets or injections, as the drug tends to stay in the bladder and very little gets into the bloodstream. We do not know how much atezolizumab, if any, will get into your bloodstream but if it does you may experience side effects in parts of the body other than the bladder.

When other types of anti-cancer drugs (e.g., BCG) are given directly into the bladder the following side effects are sometimes seen:

- Blood in the urine
- Pain on passing urine
- Needing to pass urine more often or urgently
- Inability to pass urine
- Bladder spasms or cramps
- Urine infections

These side effects are normally mild and often settle within 1-2 days of having the treatment. If they go on for longer or are severe your doctor may need to give you other medications to help with your symptoms or, rarely, admit you to hospital for further treatment.

Below are the potential side effects of atezolizumab when given via an intravenous infusion (directly into the bloodstream). We do not expect that you will have all of these side effects and we cannot predict which ones you will experience or how severe or serious they may be. Some of these side effects may be severe or fatal.

Very common: may affect more than 1 in 10 people

- fever
- nausea
- vomiting
- feeling very tired with no energy (fatigue)
- lack of energy
- itching of the skin
- diarrhoea
- joint pain
- rash
- loss of appetite
- shortness of breath
- urinary tract infection
- back pain
- cough
- headache

Common: may affect up to 1 in 10 people

- inflammation of the lungs (pneumonitis)
- low oxygen levels, which may cause shortness of breath as a consequence of inflamed lungs (hypoxia)
- stomach pain
- pain in the muscles and bones
- inflammation of the liver (hepatitis)
- elevated liver enzymes (shown in tests), which may be a sign of an inflamed liver
- difficulty swallowing (dysphagia)
- blood tests showing low levels of potassium (hypokalaemia) or sodium (hyponatremia)
- low blood pressure (hypotension)
- underactive thyroid gland (hypothyroidism)
- allergic reaction (infusion-related reaction, hypersensitivity or anaphylaxis)
- flu-like illness
- chills
- inflammation of the intestines (colitis)
- low platelet count, which may make you more likely to bruise or bleed (thrombocytopaenia)
- high blood sugar (hyperglycaemia)
- common cold (nasopharyngitis)
- mouth and throat pain
- dry mouth
- dry skin
- abnormal kidney test (possible kidney damage)
- overactive thyroid gland (hyperthyroidism)
- disorders of the membrane that surrounds the heart (pericardial disorders)

Uncommon: may affect up to 1 in 100 people

- inflammation of the pancreas (pancreatitis)
- numbness or paralysis, which may be signs of Guillain-Barré syndrome
- inflammation of the membrane around the spinal cord and brain
- low levels of adrenal hormones
- type 1 diabetes
- inflammation of muscles (myositis)
- red, dry, scaly patches of thickened skin (psoriasis)
- inflammation of the kidneys (nephritis)
- itching, skin blistering, peeling or sores, and/or ulcers in the mouth or in lining of nose, throat, or genital area which can be severe (severe skin reactions)
- inflammation of the pituitary gland situated at the base of the brain (hypophysitis)

Rare: may affect up to 1 in 1,000 people

- inflammation of the heart muscle (myocarditis)
- muscle weakness (myasthenic syndrome)
- weakness in the muscles which control facial expression (facial paresis)
- inflammation of the spinal cord (myelitis)

- inflammation of the eye (uveitis)
- red skin patches and blisters (pemphigoid)
- coeliac disease; a condition where your immune system attacks your own tissues when you eat gluten
- haemophagocytic lymphohistiocytosis; a rare disease which causes some white blood cells (histiocytes and lymphocytes) to attack other blood cells.

Other side effects that have been reported (frequency not known):

- inflammation of the bladder; signs and symptoms may include frequent and/or painful urination, urge to pass urine, blood in urine, pain or pressure in lower abdomen.

Possible risks associated with passive instillation of atezolizumab

In order to receive the drug directly into the bladder you will need to have a urinary catheter inserted each time you come for study treatment. A doctor or nurse trained to perform this procedure will insert the catheter but there are some possible risks:

- Pain – you may experience some discomfort whilst the catheter tube is being passed into the urethra. A local anaesthetic gel can be used to minimise this. Some people experience bladder spasms or cramps when they have a catheter in their bladder. If you experience this your doctor may be able to give you pain killers if needed.
- Infection – it is possible for a catheter to allow bacteria to enter the body causing infection although as the catheter will only be left in for a short time this risk is low. To avoid this, the catheter is put in using a sterile technique.
- Other less common problems include injury to the urethra (the tube that carries urine out of the body) when the catheter is inserted and narrowing of the urethra because of scar tissue caused by repeat catheter use.

Possible risks associated direct injection of atezolizumab

In order to receive the injection, you will need to have a small telescope (cystoscope) inserted into your bladder through your urethra each time you come for study treatment. A doctor trained to perform this procedure will insert the cystoscope but there are some possible risks:

- Pain and discomfort during or after the procedure
- Mild burning on passing urine for 24 hours after the procedure
- Bleeding in the urine for 1 - 3 days after the procedure
- Use of a catheter immediately after the injection to enable passing urine after treatment This happens in less than 10% of people and is usually removed within 3 days.

- Difficulty passing urine after the procedure which may require intermittent self-catheterisation
- Infection of the bladder requiring antibiotic treatment
- Recurrent urinary tract infections

Possible risks associated with other study procedures

Some of the other study procedures may have possible side effects, risks and discomforts. You may experience none, some, or all. During the study, you will, have blood taken, have CT scans, and potentially have optional MRI-PET scans. Risks of these procedures are noted below, however these are standard and routine procedures and your study doctor will be able to explain these to you in more detail and discuss any risks and/or side effects associated with these.

Radical cystectomy

The surgery is not specific to the trial and the risks will be explained by your doctor.

Drawing blood

Risks associated with drawing blood from your arm include pain, bruising, light-headedness, fainting, and on rare occasions, infection.

CT scan

CT scans use ionising radiation to form images of your body to provide your doctor with clinical information to enable you to be appropriately treated. For information about ionising radiation, please see the section titled **"Exposure to ionising radiation"** below.

A CT examination involves you having a contrast dye injected into a vein. As a result, you may experience a slight burning feeling at the injection site, a metallic taste in your mouth and hot flushes. Very rarely an allergic reaction can appear as a result of a contrast dye injected during the scan. Such allergic reaction can involve itching, rash, or in severe cases difficulty in breathing and lowering of blood pressure. If you have known of any allergic reaction to imaging contrast dyes you should let your study doctor or radiologist know.

MRI-PET

If you agree to, you may have two optional MRI-PET scans as part of the study, one before you start your study treatment, and one after your study treatment is finished, but before your bladder removal surgery. Please see **Appendix 2 – Magnetic resonance imaging-positron emission tomography (MRI-PET) sub-study** for the full details of the MRI-PET sub-study, including details of possible risks with MRI-PET.

Pregnancy and contraception during study treatment

Atezolizumab is potentially harmful to unborn children. For women of childbearing potential, if you are pregnant, think you may be pregnant or are planning to become pregnant then you must not take part in this study.

You also must not take part if you are breast feeding.

Also, for women of childbearing potential, if you join the study you will need to use a highly effective form of contraception or completely abstain from any sexual activity that could lead to a pregnancy. You would need to do this throughout your participation in the study and for at least 5 months after your last dose of study treatment. It is recommended that double methods of contraception are used.

Effective contraception in this study is defined as;

Non-hormonal methods

- Vasectomised partner (provided the vasectomised partner is the sole sexual partner of the woman of childbearing potential and that the vasectomised partner has received medical assessment of the surgical success)
- Sexual abstinence (i.e., refrain from any form of penetrative sexual intercourse between a man and a woman. Periodic abstinence (e.g., calendar ovulation, symptothermal, post-ovulation methods) and withdrawal are not acceptable methods of contraception.)
- Intrauterine device (IUD)

Hormonal methods

- Combined (oestrogen and progestogen containing) hormonal contraception associated with inhibition of ovulation:
    - Normal and low dose combined oral pills
    - Intravaginal device (e.g., ethinylestradiol and etonogestrel)
    - Norelgestromin/ethinylestradiol transdermal system
- Progestogen-only hormonal contraception associated with inhibition of ovulation:
    - Cerazette (desogestrel)
    - Hormonal shot or injection (e.g., Depo-Provera)
    - Etonogestrel implants (e.g., Implanon®, Norplant®)
- Intrauterine system (IUS) device (e.g., levonorgestrel-releasing intrauterine system e.g., Mirena®)

Your doctor or nurse will be able to advise the best contraception for you.

If you are a woman of childbearing potential you will need a negative pregnancy test in order to be eligible to take part in the study. If you do take part in the study, further pregnancy tests will also be taken every other week once you have started study treatment.

If you become pregnant during the study treatment, or at any time within the following 5 months after your last dose of study treatment, you must tell your doctor immediately. If you are still receiving study treatment, this will be stopped. Your doctor will be able to advise you on the risks to your unborn child and discuss the pregnancy with you.

If you do become pregnant during your participation in the study, we will be obliged to collect some information about you and the child and their health. The information we collect will not identify the child. We need to do this so that organisations that look after how medicines are used (such as the Medicines and Healthcare products Regulatory Agency, MHRA) have the most up-to-date information about how the study treatment affects pregnant women and unborn children.

If you do not join the study, it is still important to tell your clinical care team if you are pregnant or become pregnant as this may affect your care.

Exposure to ionising radiation

If you take part in this study you will have up to three CT scans of the chest, abdomen and pelvis. One of these will be extra to those that you would have if you did not take part. These procedures use ionising radiation to form images of your body and provide your doctor with other clinical information.

You will also have a CT urogram/intravenous urogram (IVU), following your surgery. This would happen following your surgery, regardless of your participation in the study.

Ionising radiation may cause cancer many years or decades after the exposure. The chances of this happening to you as a consequence of taking part in this study are about 0.5%.

For those participating in the sub-study, you will have two PET MR scans in addition to the CT scans. Both of the PET MR scans will be extra to those that you would have if you did not take part. These procedures use ionising radiation to form images of your body and provide your doctor with other clinical information.

Ionising radiation may cause cancer many years or decades after the exposure. The chances of this happening to you as a consequence of taking part in both the study and the sub study are about 0.6%.

How is my condition monitored?

Within this study, your bladder and cancer will be monitored with an additional CT scan. Your response to the study treatment will be monitored with blood and urine tests and regular contact with your doctors and nurses to check any side effects or complications you may experience. Your bladder removal surgery is planned within the next two months. After surgery we will see you regularly in outpatients to check your recovery and to see if you need any further treatment.

What are the possible disadvantages and risks of taking part?

All treatment for cancer involves some risks as well as potential benefits. The kind of side effects that you may have from this drug and the study procedures involved are listed above in the section **"Might the study treatment have any unwanted effects?"**. As this study is investigating new methods of administering the drug, we believe that these

side effects may be less likely. Previous patients who have been treated with this drug have had it administered via intravenous infusion, whereas this study is administering it directly into the bladder. This may reduce the chance of experiencing side effects that affect areas other than the bladder.

In addition, the study treatment methods (passive instillation and direct injection) are well established. However, as very few patients have received atezolizumab via one of these treatment methods before, there is a chance that there may be side effects which are new, or which differ from those which doctors would usually expect to see. The potential risks and side effects associated with your treatment will be discussed in detail with your doctor.

What are the possible benefits of taking part?

There is no guarantee that you will benefit from the study treatment given in this study, but you will still receive the same cancer treatment (surgery to remove your bladder) whether or not you take part in the study. Also, your surgery will not be delayed by going into the study as the study treatments with atezolizumab will be fitted in before your planned surgery. It is not yet known whether atezolizumab treatment will work for localised bladder cancer. If it does work, it is hoped that it may treat the cancer and stop it from coming back or spreading into the deeper layers of the bladder. However, this cannot be guaranteed and if the drug does not work you may not get any benefit from the study treatment. Any benefits from taking part in the study may only be temporary.

Therefore, your participation in this study may not be of direct benefit to you personally, but it is possible that it may be of benefit to future cancer patients. Information from this study will help doctors to learn more about atezolizumab when it is administered directly in the bladder and whether this may be a step forward for the treatment of bladder cancer in the future. Without research of this sort, improvements in cancer treatments are not possible. All study participants could benefit from more close monitoring than would be possible outside of the study.

Will I get back any travel or other costs?

Reimbursement is available for travel costs incurred because of visits you undertake specifically to take part in the study (e.g., visits for study treatment). Any additional hospital visits will be minimised by collecting your data where possible at times when you would already be visiting hospital outside of the study, and an expense limit will apply. Please talk to your doctor or nurse in the study team to arrange reimbursement for these expenses.

What if something goes wrong?

As with any treatment, your doctor will aim to ensure that any risks are kept to a minimum. The study has an independent Safety Review Committee which will closely monitor the study on an on-going basis, so that if there

are any problems then they will be detected as soon as possible so that the study can be changed or stopped if necessary. If you experience any problems after the start of study treatment, you must report these to your study nurse or doctor.

We do not expect anything to go wrong, but if a medical emergency related to your treatment for this study occurs while you are at home, you should go to the Accident & Emergency (A&E) department at your local hospital. If you are unable to get to the hospital you should call 999. You will be provided with a study ID card. Please use the contact details provided on the card to get in touch with the research team to inform them if you have attended hospital. You can also show this ID card to your doctor to provide contact details for the research team and details of the study drug.

What mental health support is available to me?

Specialist nurse support and access to clinical psychology services is available as part of your cancer care. There are also additional services, both within the NHS and through charities, which can also offer support. Please contact your research nurse (contact details available on the first page), who can signpost you to the relevant services. There are also contact details below for Macmillan Cancer support.

What happens when the research study stops?

Study treatment will be for a maximum of 6 weeks prior to the removal of your bladder. You will undergo bladder removal surgery and receive standard follow-up care for that procedure. Additional data will be collected at 2 years after the final participant enters the study. You will not be contacted to collect this data; this data will be obtained from your healthcare records.

In the unlikely event that the study ends early due to unforeseen circumstances while you are still receiving study treatment, you will stop study treatment and will have your surgery as per the standard treatment outside of the study. You will still be monitored for any side effects.

Contact details

If you have any further questions about your illness or clinical studies, please discuss them with your doctor or research nurse (see first page for contact details).

You may also find it helpful to contact Macmillan Cancer Support, an independent cancer information charity (freephone: **0808 808 00 00**; address: **89 Albert Embankment, London, SE1 7UQ;** website www.macmillan.org.uk) or get more information from the charity Cancer Research UK at https://www.cancerresearchuk.org/about-cancer. If you would like further information about clinical research, the UK Clinical Research Collaboration (a partnership of organisations working together on clinical research in the UK) have published various resources to help people learn more about

clinical trials. Contact UKCRC: Tel: **0207 395 2271**; email: info@ukcrc.org; website www.ukcrc.org.

This is the end of **Part 1** of the Information Sheet. If the Information in **Part 1** has interested you, please continue to read the additional information in **Parts 2 and 3** before making a decision about taking part.

Part 2 – more about this study

What if relevant new information becomes available?

Sometimes during a study, new information becomes available. If this happens your doctor will tell you about it and discuss with you whether you want to continue in the study. If you decide not to continue, your doctor will continue your care if this is necessary. If you decide to continue, you may be asked to sign an updated consent form. Occasionally on receiving new information, your doctor may consider it to be in your best interests that you withdraw from any further study treatment.

What will happen if I don't want to carry on with the study?

You can stop taking part in this study, or in any part of it, at any time and without giving a reason. However, we would like to know the reason, if you are willing to say, because this can be useful when we produce the results of the study.

Before deciding to stop, you should talk to your study doctor or nurse. They can advise you and may be able to deal with any concerns you may have. If you decide to stop taking part at any time, it will not affect the standard of care you receive.

If you decide to stop receiving your study treatment, you will receive your bladder removal surgery as planned. Study visits and assessments can still go ahead if you agree to this.

If you decide to stop study visits or assessments, to make sure the research is still reliable, we will need to keep the information we have already collected about you and include it in the study analysis.

Unless you clearly tell us you don't want us to, we will continue collecting information about your health from routine hospital visits, via your GP or through other contact between you and your hospital. We will only do this if the information is relevant to the study. We do this to help ensure the results of the study are valid. You can read more about this in **Part 3** of this information sheet.

Unless you clearly tell us you don't want us to, we will keep any biological samples (such as blood samples, tissue biopsies and urine samples) you have given for future research purposes.

Finally, we may be legally required to collect information about any side effects you have following your study treatment, even if you have told us you did not want to provide further data for the study.

Will my taking part be kept confidential?

There are a few things you should know about how your confidentiality may be affected if you agree to take part in this study.

- Your **GP, and the other doctors involved in your healthcare, will be kept informed** of your participation in this study. This is because they might need to know you took part when they treat you for anything in future.

- We may also **need to contact your GP and other doctors** involved in your healthcare if you have not had any study visits for a while, to check you are still OK to take part in the study.
- Your **healthcare records may be looked at by authorised individuals** from the research team, Leeds Clinical Trials Research Unit, Sheffield Teaching Hospitals NHS Foundation Trust (the study Sponsor), the regulatory authorities or other authorised bodies to check that the study is being carried out correctly. This will only be done in line with your hospital's policies to ensure your records are secure.
- Your study records **will be regularly reviewed by the Safety Review Committee,** to monitor the safety of study treatment and check that the study is being carried out correctly.
- Your study records **may be inspected by authorised individuals from F. Hoffmann-La Roche Ltd (the study Funder)** to monitor the safety of study treatment and check that the study is being carried out correctly.
- Your study records **may be passed to the research team, Leeds Clinical Trials Research Unit, Sheffield Teaching Hospitals NHS Foundation Trust (the study Sponsor), and F. Hoffmann-La Roche Ltd**. for the purpose of analysing the data, publishing the results, and carrying out further research.
- Leeds Clinical Trials Research Unit would like to **collect a copy of your completed consent form** if you agree to take part in the study. This is so that we can check you have definitely agreed to take part. This means people in the study team who are authorised to deal with consent forms will see your name. However, these people are trained to treat your information with care, and the consent form will be stored securely at all times.
- The **biological samples (blood and urine) taken from you in this study for PK/ADA analysis will be sent to ICON PLC laboratory, located in the USA.** The staff at the lab will be bound by their professional responsibilities to keep all your information private and confidential. The Sponsor will contract with the Lab to ensure information about you is kept safe & secure. The samples themselves will be labelled with a unique study ID and your year of birth, and not your name.
- Biological samples (tissue, blood and urine) taken from you for future translational research will be collected and processed by trained staff and stored in a **Human Tissue Authority (HTA) Licensed storage facility at the Sheffield Biorepository**. The staff at the lab will be bound by their professional responsibilities to keep all your information private and confidential. The samples will be

stored, used and shared in a way that the researcher is not able to identify you (anonymised).

- In this study, we will collect MRI-PET scans if you consent to take part in the MRI-PET sub-study. These will have any details that could identify you removed before they are sent, securely, to **University of Sheffield**, who will carry out this analysis.

Who has organised, reviewed and funded the research and who will be supervising it?

Sheffield Teaching Hospitals NHS Foundation Trust is the sponsor and has reviewed and approved this study and will be responsible for the management and conduct of the whole study.

The day-to-day running of this study will be carried out by the Clinical Trials Research Unit (CTRU) at the University of Leeds.

Pharmaceutical company F. Hoffmann-La Roche Ltd. are funding the research and providing the atezolizumab for use in the study. Roche have been involved in developing the study and will continue to provide safety information on atezolizumab during the study.

All research in the NHS is looked at by an independent group of people, called a Research Ethics Committee, to protect the safety, rights, wellbeing and dignity of any participants that may take part. The study and associated documentation has been reviewed and approved by the London Central Committee.

The project has also received approval by the Health Research Authority (HRA) and Medicines and Healthcare products Regulatory Agency (MHRA)

The study has a Safety Review Committee which will closely monitor the study on an on-going basis, so that if there are any problems then they will be detected as soon as possible so that the study can be changed or stopped if necessary. If you experience problems, you must report these to your study nurse or doctor.

How can I raise a complaint about something that has happened in the study?

If you wish to complain or have concerns about any aspect of the way you have been approached or treated during the course of this study the normal NHS complaints mechanisms should be available to you. Your doctor will give you further information if necessary.

What if I am harmed by taking part in the study?

Every care will be taken in the course of this clinical study. However, in the unlikely event that you are injured as a result of the Sponsor organisation (Sheffield Teaching Hospitals NHS Foundation Trust), provides NHS indemnity for harm caused by the negligent actions of its employees, through its membership of the Clinical Negligence Scheme for Trusts (CNST). The Trust is not able to provide "no fault" indemnity for non-negligent harm.

As employers of the authors, University of Sheffield and University of Leeds the provide indemnity to cover negligence only liabilities arising from the design of the research.

Your hospital where you receive your study treatment has a duty of care to you whether or not you agree to participate in the study and the University of Leeds accepts no liability for negligence on the part of your hospital's employees. If you wish to complain about any aspect of the way you have been treated please contact your research doctor in the first instance.

Any claims will be subject to UK law and must be brought in the UK.

If you have private medical insurance, you should tell your insurer that you are taking part in research. They will let you know if it affects your policy.

What will happen to any blood, urine or tissue samples I give?

Blood and urine samples for PK/ADA

Blood **and** urine samples will be taken as part of the trial, to analyse pharmacokinetics (PK) and anti-drug antibodies (ADAs). These samples will be sent to laboratories outside your hospital contracted by the sponsor (ICON PLC laboratory, located in the USA). These will be labelled with your study number and year of birth; they will not contain your name and address. These samples will be used to inform our understanding of which patients respond to therapy and the underlying mechanisms of response to study treatment.

These samples will be taken on the first day of study treatment, both immediately prior to study treatment and at intervals following your first dose of study treatment. If you are allocated to receive multiple doses of study treatment, each week blood and urine samples will be taken immediately prior to each dose, and 0-2 hours immediately after receiving study treatment. All participants will also have samples taken approximately 1 week following your final dose of study treatment. You will be asked to donate up to an extra 7-14 ml of blood (approximately 0.5-1 tablespoons) on each occasion. This is a safe amount of blood to take, and you will not be harmed.

All samples will be sent to the laboratories by a specialist courier and will be labelled in such a way that the recipient cannot identify you (anonymised).

When your samples are sent to the laboratories for further tests, there may be some of your samples left over once all the tests have been done. These will be kept in case they need to be re-analysed for quality control purposes. Any remaining samples will be destroyed at the end of this study.

Additional biological samples

You will also be asked to donate additional samples of blood, urine, and tissue from your tumour and bladder for future translational research, which may include genetic testing and the transfer of your samples to other organisations, including commercial companies, and organisations based outside of the UK. This additional research will only

be undertaken after approval has been received from an ethics committee. The results of these tests may be published in scientific journals, but you will not be identified from these.

These samples will be collected and processed by trained staff and stored in a Human Tissue Authority (HTA) Licensed storage facility at the Sheffield Biorepository. The staff at the lab will be bound by their professional responsibilities to keep all your information private and confidential.

All samples will be sent to the Biorepository using your hospital's standard processes or by a courier.

Extra blood samples

You will be asked to donate whole blood, serum and plasma samples for this research, which will be collected prior to each dose of study treatment, and just before your bladder removal surgery, and a plasma sample approximately 4 or 8 weeks after your surgery.

These will be taken at the same time as your normal blood tests wherever possible; you will be asked to donate up to an extra 20-70 ml blood (approximately 1.5-4.5 tablespoons) on each occasion. This is a safe amount of blood to take, and you will not be harmed.

Extra urine samples

You will also be asked to donate urine samples for this research, which will be collected at the start of the study, prior to each dose of study treatment, just before your bladder removal surgery, and approximately 4 or 8 weeks after your surgery.

Donation of surplus tissue from biopsies

You will not need to have any additional biopsies specifically for the study. However, you will also be asked to donate tissue samples, which will be taken from the biopsy done to confirm your diagnosis of bladder cancer, and from the bladder as it is removed during surgery. We will always make sure some tissue remains available to the hospital pathology team; in case it is needed for your future care.

Will any genetic tests be done?

Genetic and other molecular biology tests may be done on the blood, urine and tissue samples you provide. For example, tests may be performed to assess whether a person's genetic make-up can tell us in advance whether they are more or less likely to benefit from the drug used in this study in the future, or whether they are more or less likely to experience side effects. You will not be told the results of any of these tests performed on your samples.

<u>What will happen to the results of the research study?</u>

When the study is complete the results will be published in a medical journal, but no individual participants will be identified. We will make sure you have a chance to find out the results of the study if you would like them.

The results of the research will be presented at academic meetings and in the medical literature. Your hospital doctor and the wider medical community will be notified of any information that would change current medical practices and improve patient treatments and outcomes.

Part 3 – how we will use your information

If you decide to take part in this study, some information about you will need to be collected and used. This section explains what information will be collected, who it will be shared with and what it means for you.

We know that some people want to know more than others about how their information is used. You can therefore choose how much detail you'd like:

- You can look at the **quick access guide**, below. You should definitely read this, even if you do not look at the appendix and comprehensive guide mentioned below.
- If you have particular questions or concerns, you should look at the **optional appendix**. This is available at the end of this information sheet.
- If you would like more detailed explanations about anything, including why we need to do things in a certain way, you can find it in our **comprehensive guide**. You do not have to read it before taking part in this study, but you might want to look at specific sections if you have particular concerns. This is available at https://ctru.leeds.ac.uk/ctru-comprehensive-privacy-guide/ or you can ask for a printed copy from your study doctor or nurse**.** This can also be made available in large print or other formats if you need them.

The text in each of these documents is laid out in the same order, so you can easily find more detail.

All of these documents have been written with the help of patients and the public to help make sure they are clear and accessible. As in the rest of this participant information sheet, whenever we say 'we' or 'us', we mean the study team at the Clinical Trials Research Unit, University of Leeds (https://ctru.leeds.ac.uk/).

You can find more general information from the NHS about how people's information is used in research at https://www.hra.nhs.uk/information-about-patients/.

A quick access guide to how we will use your information in INVEST

You can read more about each of the points below in the optional appendix below. **Use the reference numbers in brackets to find the relevant section in the optional appendix.**

- If you agree to take part in this study, we will collect information about you and your health. **(1)** We will use this to run the study, produce the study results, and to help make sure you and other people taking part in the study are safe. The information we will collect will include:
  - Information from you and from your hospital medical notes
  - Information from analysing biological samples you give (e.g., blood, urine and tissue samples)
  - Images from CT scans, MRI-PET scans (optional), IVU scans
- Your information will be collected by the Clinical Trials Research Unit (CTRU) at University of Leeds (https://ctru.leeds.ac.uk/), who are running this study. **(2)**
- We will keep all your information secure at all times. **(3)** The only people at the CTRU at University of Leeds who will see your information are the people who need to run or analyse the study or check how the study has been run. **(4)**
- Sheffield Teaching Hospitals NHS Trust are the study sponsor and will have overall responsibility for how your information is used in this study, including making sure that all information is kept secure. **(2)**
- Your study records will be shared with the research team, Leeds CTRU and Sheffield Teaching Hospitals NHS Foundation Trust (the study Sponsor) for the purpose of analysing the data, publishing the results, and carrying out further research. **(4)**
- We may use the study information for additional research projects within the University of Leeds or Sheffield Teaching Hospitals NHS Trust. We will only do this for worthwhile research projects with all appropriate ethical approvals. If people outside the original study team are involved, they will only receive the minimum information needed for the new project, and they will not receive any clearly identifiable information (such as your name). **(5)**
- We will sometimes need to share your information with people outside the CTRU at University of Leeds or Sheffield Teaching Hospitals NHS Trust. This is so that we can run the study, keep you and others safe, comply with laws and other rules around research, and support further research in the public interest. We will never sell your information or pass it on to people who will sell it. Information that we share will never be

used to make decisions about future services available to you, such as insurance. **(5)**

- F. Hoffmann-La Roche Ltd. who are funding this research, have the right to audit the trial for purposes of monitoring the safety of study treatment and check that the study is being carried out correctly. We may share the anonymous Data with F. Hoffmann-La Roche Ltd to add to their data pool on the drug. **(5)**
- We will share your data and biological samples with ICON PLC laboratory, located in the USA for the purposes of the PK/ADA work. We will share only limited data (your year of birth and study number, to ensure we identify who each sample belongs to). **(5)**
- In this study, if you consent to the optional MRI-PET scans, we will collect two MRI-PET scan reports. These will have any details that could identify you removed before they are sent to us. These scans will be securely provided to the University of Sheffield, who will carry out analysis. **(5)**
- You can usually ask organisations to give you a copy of information they hold about you, or to correct your information. However, this does not apply when your information is used for research in the public interest like this, because allowing you to access or change the information could harm the quality of this research. **(6)**
- To comply with laws and other rules about research, we need to keep your identifiable information until at least 15 years after the study has finished. **(7)**
- You can usually ask organisations to delete your information or restrict how your information is used. However, allowing you to delete or restrict your information could harm the quality of this research, which is being done in the public interest. If you stop taking part in the study, we will therefore need to keep the information we already have about you. **(8)**
- If you tell us you want us to stop collecting your information, we will still be legally required to collect information about any serious side effects you may experience. **(8)**
- If you have questions or concerns about how your information is used that aren't answered by this document or by talking to your study doctor or nurse, you can contact the University of Leeds or Sheffield Teaching Hospitals NHS Foundation Trust Data Protection Officer. If you are still not happy, you can contact the Information Commissioner's Office. You can find out how to contact these people in the optional appendix and the comprehensive guide. For any questions or concerns that are not to do with how your information is used in this study, please contact your nurse or doctor as you usually would. **(9)**

Appendix 1 – Visit schedule

Assessment	Registration	Eligibility	Within 7 days between eligibility & start of study treatment	Study treatment visits			Follow-up visits						Follow-up data sweep
				Up to 3 days before starting study treatment	Up to 3 days before continuing study treatment (every week)	On day of study treatment (every week)	1 week after finishing study treatment, before surgery	At time of surgery	7-day hospital stay post-surgery	4 weeks post-surgery	8 weeks post-surgery	12 weeks post-surgery	2-year sweep
Signing consent form	x												
Collection of demographic information and bladder cancer information	x												
Collection of medical history		x											
Physical examination		x	x*	x*	x*		x*	x*	x*	x*	x*	x	
Electrocardiogram (ECG)		x	x*	x*	x*		x*	x*	x*	x*	x*	x*	
CT scan (chest, abdomen & pelvis)		x					x					x	
CT urogram/Intravenous urogram (IVU)										x			
Pregnancy test (if applicable)		x		x	x						x		
Safety blood/urine tests		x		x	x			x		x	x	x	
Discussion with doctor about symptoms, side-effects and medicines you are taking			x	x	x	x	x	x	x	x	x	x	
MRI-PET scan (bladder) (optional)			x				x						
Tissue samples for future translational research		x						x					
Blood & urine samples for future translational research						x	x			x	x^		
Blood and urine samples for PK/ADA						x	x						
Collection of surgery details & complications								x	x	x	x		
Clinic appointment to review surgery recovery											x	x	
Survival and cancer status data sweep													x

**If your doctor thinks this assessment is medically necessary*

^If not already taken at 4-week follow-up visit

Appendix 2 – Magnetic resonance imaging-positron emission tomography (MRI-PET) sub-study

At registration, you will be asked if you are willing to have an optional magnetic resonance imaging-positron emission tomography (MRI- PET) scan of your bladder. These scans are entirely optional, and if you decide not to take part, this will not affect your participation in the INVEST trial as a whole.

These scans will be taken at the following times:

- after you have been confirmed as eligible to take part but before study treatment, and;
- following the end of study treatment but prior to bladder removal surgery.

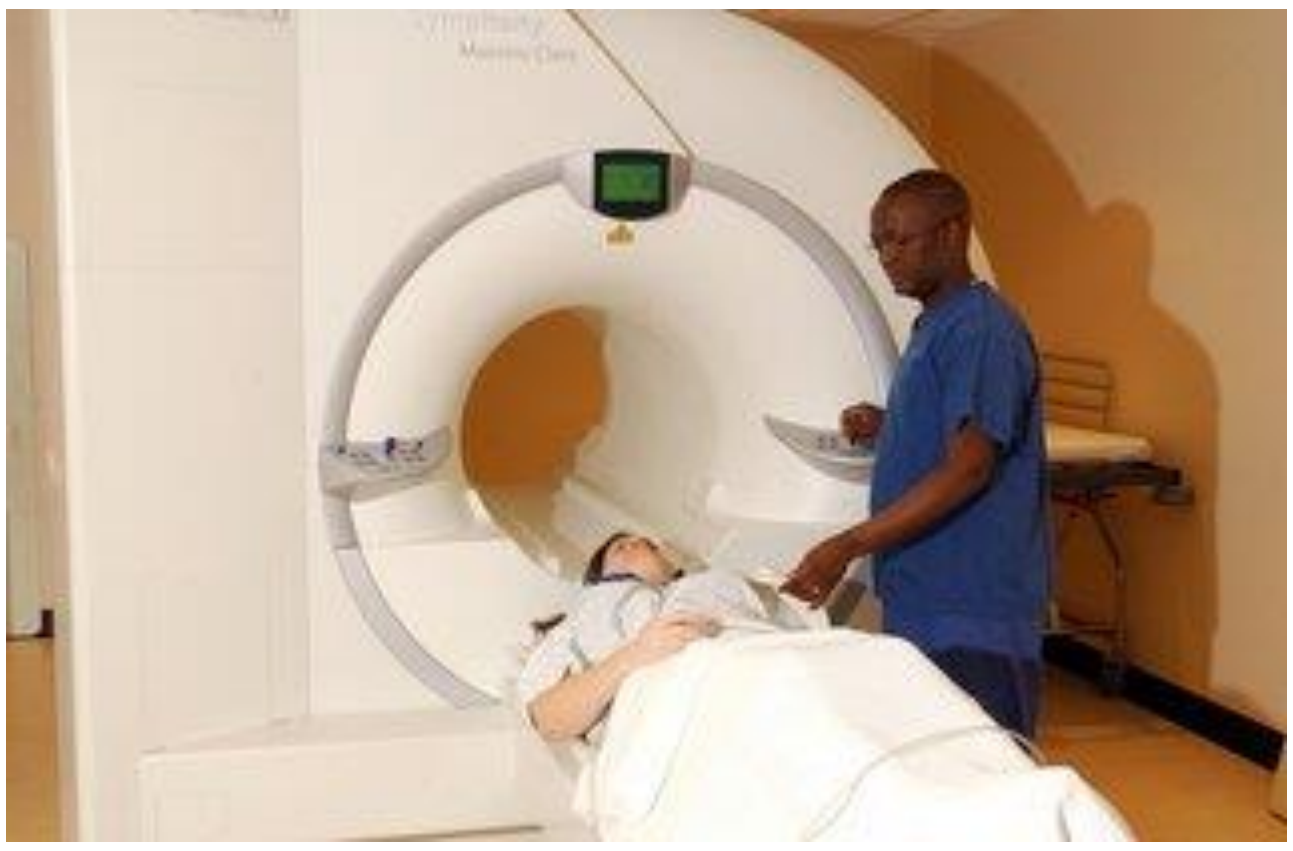

3 - https://www.nhs.uk/conditions/mri-scan/

The following will happen at each scan if you agree to take part. In total, you can expect to be in the scanning department for at least 3 hours each time. You will be asked to prepare for the scans by fasting for 6 hours prior to your appointment and will have a blood glucose check prior to the scans. You will also be asked to be well-hydrated in advance of your appointment. You will be asked to drink 500ml of water immediately prior to receiving an injection of FDG tracer (radiotracer). This will take place on the scanner, and the initial MRI-PET scan will be completed immediately after receiving this injection. This scan will take approximately 10 minutes.

Following this, there will be a period of about 50 minutes where you will be asked to rest in a separate room. During this period, you will be asked to void your bladder as much as possible. After the 50 minutes, you will be taken back to the scanner for some MRI scans as well as a further MRI-PET scan. These scans will take a total of about 1 hour 20 minutes. During this period of scanning, you will be encouraged not to use the toilet again. You will be injected with an MRI contrast agent part way through the MR scans.

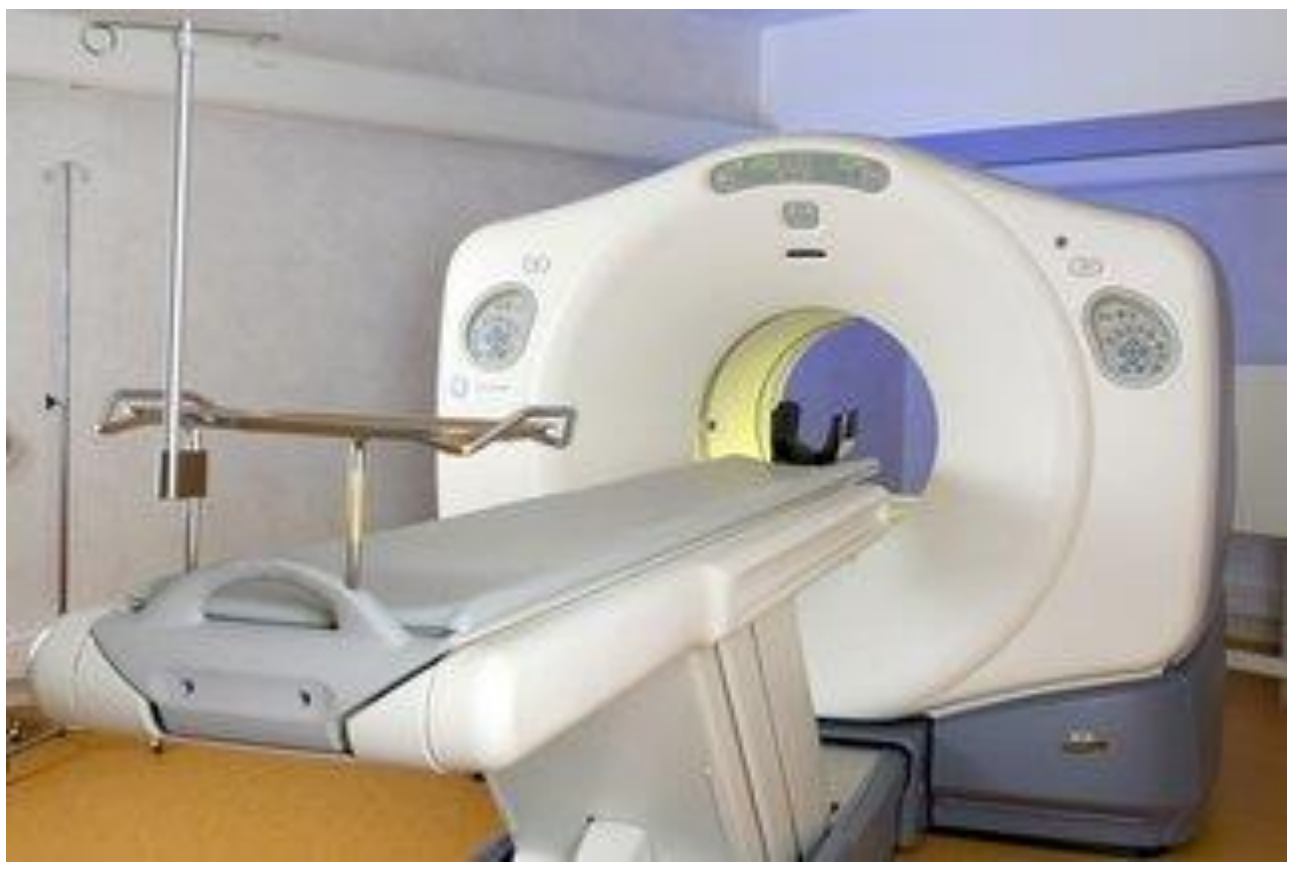

4 - https://www.nhs.uk/conditions/pet-scan/

These scans will have any details that could identify you removed before they are sent to us. They will be securely provided to University of Sheffield and Sheffield Teaching Hospitals, who will carry out this analysis to see if there appears to be an early response to the trial treatment.

Potential risks of the MRI-PET scan

MRI-PET scans use a mildly radioactive drug to show up areas of your body where cells are more active than normal, in combination with magnetism and radio waves to create cross section pictures of the body. For information about ionising radiation, please see the section titled **"Exposure to ionising radiation"**.

An MRI-PET scan involves you having a radiotracer injected into a vein. A very small amount of radioactive tracer is left in your body for a short time after your scan. So, for the rest of the day keep any time you spend within arm's length of pregnant women, babies or young children as short as possible. Your radiographer will advise you about this.

You will also have an MRI contrast dye injected into a vein. As a result, you may experience a slight burning feeling at the injection site, a metallic taste in your mouth and hot flushes. Very rarely an allergic reaction can appear as a result of a contrast dye injected during the scan. Such allergic reaction can involve itching, rash, or in severe cases difficulty in breathing and lowering of blood pressure. If you have known of any allergic reaction to imaging contrast dyes you should let your study doctor or radiologist know.

There is a risk that the radioactive tracer or contrast will leak outside the vein. This can cause swelling and pain in your arm but it's rare and will get better quickly. Tell your radiographer if you feel any pain or swelling around the site. You might get a small bruise around the area where your radiographer puts the cannula in.

You may also experience some claustrophobia while having these scans. If you feel you may experience claustrophobia, please let the department staff know before your appointment. They will be able to take the time to make sure you understand the procedure and make sure you are comfortable. Your doctor can also give you medicine to help you relax if you feel this would be beneficial.

It is not expected, but it is possible that these scans may discover something about your health that you and your clinicians are currently unaware of, for example that your cancer has spread further than already known (within the limited range of the PET MR scan). In such circumstances the result will be discussed with your cancer care team as soon as this finding is identified, who will then explain the appropriate treatment options.

Optional appendix – more about how your information will be used

This optional extra section of this participant information sheet is about how your information will be used if you agree to take part in this study. It gives you more detail than the **quick access guide** above. It is in the same order as that, so that you can easily find what you need. If you have questions or concerns after reading the quick access guide, you should look at this optional appendix, or the sections of it that interest you.

If you still have questions after reading this appendix, or would like more detail about anything, you should look through our **comprehensive guide** to how your information is used. You do not have to read it before taking part in this study, but you might want to look at specific sections if you have particular concerns. It is available at https://ctru.leeds.ac.uk/ctru-comprehensive-privacy-guide/ or you can ask for a printed copy from your study doctor or nurse. This can also be made available in large print or other formats if you need them.

You should read through these sections as much as you would like to. After doing that, if you are interested in participating in the study, you can find the informed consent form at the end of this document.

1. What information will be collected, and what will it be used for?

If you agree to take part in this study, we will need to collect and use some information about you and your health. This research is in the public interest, which means our results will be used to improve the healthcare of patients in the future. Because of this, if you agree to take part in the study, we will be able to use information about you, including sensitive information about your health.

This information will include the following;

- Initials
- NHS number
- Name
- Date of birth
- Ethnicity
- Sex
- Medical history
- Your genetic characteristics (this means information about your DNA from biological samples you will have given).

We will only use what we need to run the study, to produce the results of the study and to make sure you and other people taking part in the study are safe.

Specifically, the ways we will use information about you are:

- We will use information from you and your medical notes to run the study, to produce the study results and to confirm it is safe and appropriate for you to join the study. We will also collect information about your health from your study doctor or nurse and your medical notes to help make sure you and others are safe.
- We will collect a copy of your signed consent form so that we can be sure you have agreed to take part in the study.
- We will collect information about biological samples (blood, urine and tissue samples) you will give in the study. We will provide these biological samples to the central laboratories as detailed below. These samples will only include your study number and year of birth. In addition to these it may become necessary to use alternative central laboratories during the study. This data will be shared with the research team, the study sponsor and F. Hoffmann-La Roche Ltd.
    - The biological samples (blood and urine) taken from you in this study for PK/ADA analysis will be sent to ICON PLC laboratory, located in the USA. The staff at the lab will be bound by their professional responsibilities to keep all your information private and confidential.
    - Biological samples (tissue, blood and urine) taken from you for future translational research (once INVEST has been completed) will be collected and processed by trained staff and stored in a Human Tissue Authority (HTA) Licensed storage facility at the Sheffield Biorepository. The staff at the lab will be bound by their professional responsibilities to keep all your information private and confidential.
    - If other researchers in the future want to use the samples for research in the public interest, they will be sent the samples and a unique identification number. The other researchers may also ask us for other information about you for linkage to their research, but they would not be able to see who you are from the information they have, even when they combine the results of analysing your samples with the other information we share with them.
- Sometimes we need to ask doctors who work with us to give us advice on specific medical situations. To help the doctors do this, we might need to collect copies or scans of parts of your medical notes. These will have any details that could identify you removed before they are sent to us.
- In this study, if you consent to the optional MRI-PET scans, we will collect two MRI-PET scans. These will have any details that could identify you removed before they are sent to us. These scans will be securely provided to the University of Sheffield, who will carry out analysis.

If you want to find out more about any of these, please refer to the **comprehensive guide** to how your information is used.

2. Who is collecting my information?

University of Leeds and Sheffield Teaching Hospitals NHS Foundation Trust, who are the 'sponsor' of the study, will act as "Data Controllers". This means they jointly have responsibility for what information is collected, how it is collected, and making sure people's information is used securely and correctly. If you want to contact someone within the University of Leeds about how your information has been or will be used, you can see **section 9**, below. See the **comprehensive guide** for more about what this means for you.

Your information will be collected by the Clinical Trials Research Unit within the Leeds Institute of Clinical Trials Research, University of Leeds. You can find out more about our work at https://ctru.leeds.ac.uk/.

You can find out more about how your information will be used at https://www.sheffieldclinicalresearch.org/for-patients-public/how-is-your-information-handled-in-research/ (for Sheffield Teaching Hospitals NHS Foundation Trust).

We will make sure we follow the principles of data protection in everything we do. This means we will keep your information secure, keep it only for as long as we need it, only use the minimum information we need for specific, necessary purposes, and we will be open, transparent and fair with you about how we use your information. You can find out more about how we follow the principles of data protection in the **comprehensive guide**.

3. Will my information be kept secure?

We will take all necessary measures to ensure that information about you is sent and stored securely by us or by anyone acting on our behalf.

Your study doctor or nurse will enter most of the information needed for the study directly into our secure study database. Your study doctor or nurse may also send us some information by post. This may include your completed consent form, so that we can be sure you have agreed to take part in the study. This will be sent separately to any other study forms. The study team may access your electronic healthcare records remotely to check that the study is being carried out correctly.

Sometimes we will also get information about you by email. Emails will never contain your name, only your study identifying number and sometimes your initials and date of birth. Emails will be sent securely and will be encrypted as per NHS guidance.

Finally, some particularly sensitive documents, such as your consent form will be sent to us via a 'secure file transfer'. This means information is sent by the internet in a very secure way.

Information stored in our databases or other electronic storage locations is held very securely, in a way that would make it very difficult for any unauthorised people to access it.

4. Who will see my information in the research team?

We will make sure that the only people at the Clinical Trials Research Unit at University of Leeds who can see your information are people who need to run or analyse the study. People who do not need to know who you are will not be able to see your name or contact details. Your data will have a study number instead. See **Part 2** of this information sheet for more on who may see your medical notes and other confidential information if you agree to take part in this study.

5. Who else will see my information?

There are some specific situations where we need to share information with other people or other organisations. We will always do this carefully and only when it's necessary. We will avoid sharing information that could identify you whenever possible. We will never sell your information or pass it on to people who will sell it. We will only share information when it is necessary for the study, necessary to protect your safety or the safety of others, or in the public interest. Information we share will not be used to make decisions about future services available to you, such as insurance.

We will share your information for the following reasons. You can find out more about these in the **comprehensive guide**.

- To run and analyse parts of the study, we need to share your information with collaborators (such as doctors, statisticians or other experts) outside the University of Leeds or Sheffield Teaching Hospitals NHS Foundation Trust.
- To keep you and other people safe, we will need to share some information about health-related events you may have with authorised organisations. None of these organisations will be able to identify you from this information.
- To report to authorised people about the progress of the study, we will need to share some basic information with some authorised organisations, including the Research Ethics Committee that has approved this study. None of these organisations will be able to identify you from this information.
- To keep you and other people safe, we will need to share some information about health-related events you may have with the Safety Review committee.
- Your study records may be inspected by authorised individuals from F. Hoffmann-La Roche Ltd to monitor the safety of study treatment and check that the study is being carried

out correctly. In addition to this we may share the anonymous study data with F. Hoffmann-La Roche Ltd to add to their data pool on the drug.

- The biological samples (blood and urine) taken from you in this study for PK/ADA analysis will be sent to ICON PLC laboratory, located in the USA. These will be sent with your year of birth and study number.
- In this study, if you consent to the optional MRI-PET scans, we will collect two MRI-PET scans. These will have any details that could identify you removed before they are sent to us. These scans will be securely provided to the University of Sheffield, who will carry out analysis.
- To allow other researchers to carry out future research in the public interest. We will only share your information for worthwhile research with all appropriate approvals. We will only share your information in such a way that researchers outside the University of Leeds will not be able to identify you. Your information will not be shared if you have explicitly said you did not want this to happen.
- We may also use study information for additional research projects within the University of Leeds or Sheffield Teaching Hospitals NHS Foundation Trust. We will only agree to do this for worthwhile projects with all appropriate approvals, and we will not share any clearly identifiable information with researchers outside the original study team.
- Due to storage space limitations, we will store information securely away from the University of Leeds for a period after the main part of the study is over. The archiving companies we use to do this for us will only store your information and will not access it or see your details.

See **Part 2** of this information sheet for more on who may see your medical notes and other confidential information if you agree to take part in this study.

6. <u>Can I see my information, or ask you to correct it?</u>

Usually, when an organisation or a company has information about you, you can ask to have access to that information at any time or ask them to correct it if it needs correcting. However, this does not apply in the same way to information used for research in the public interest, because allowing people to access or change their information could harm the quality of the research. You therefore cannot ask to access or correct information we have about you. However, most of the information we will collect will also be in your medical notes, which you can get access to if you want to. You should speak to your study doctor and nurse if you would like more information about care you have received.

7. <u>How long will my information be stored for?</u>

If you agree to take part in this study, we will need to keep your information for at least 15 after the end of the study. We need to do this in order to comply with laws and other rules about research, which say it must be possible to check the results of the research for a period of time after it has finished. We will keep your information secure during all this time. Your information may be transferred to the sponsor, and this will also be kept for at least 15 years. For practical reasons, we may ask reputable archiving companies to store information securely on our behalf, away from the University of Leeds.

At the end of this period, we will securely destroy your information.

8. What will happen if I stop taking part in the study?

If you decide you would like to stop all your study visits for any reason, we will need to keep the information we have about you to make sure the results of the study are reliable.

Usually, when an organisation or a company has information about you, you can ask them to delete it, or not use it for a certain purpose. However, this does not apply in the same way to information used for research, because it would harm the quality of the research if people could delete or remove their information. We also need to comply with laws and other rules about research that say we need to keep all information used in research for a period of time after the research finishes. If you agree to take part in this study, it will therefore not be possible for us to remove or delete your information later on, although you can ask us to collect no further information after a given time.

Some other things you should know about what will happen to your information if you stop taking part:

- If you stop all study visits, you should discuss with your study doctor and nurse. If you still occasionally go to your hospital for routine visits, we would like to hear from your study doctor or nurse about these visits, if they are relevant to this study. This way, you can still contribute to the study and help make the study results more reliable, without giving any more of your time. However, you can tell your study doctor or nurse that you do not want any more information sent to us, and they will make sure your wishes are respected.
- If you tell us you do not want us to collect any more information about you, we will still be legally required to collect information about any serious side effects you experience, or health events that might be related to the study treatment you have received. This is so that doctors using the same treatment have all the information they need about possible side effects.
- If you stop attending your study visits without telling anyone at your hospital, or you change your contact details or move house and do not tell your hospital, they will lose contact with

you. If this happens, we may ask your study doctor or nurse to contact your GP to check if you are OK and still happy to take part in the study.

If you want to know more about what might happen to your information if you stop taking part in the study, including *why* we need to use your information in the ways we do, please see the **comprehensive guide**.

9. What if I have concerns about how my information is being used?

Sheffield Teaching Hospitals NHS Foundation Trust is the sponsor for this study based in the United Kingdom. University of Leeds (CTRU) is coordinating the study and is based in the United Kingdom.

These two organisations will be using information from you and your healthcare records in order to undertake this study and will act as joint data controllers for this study. This means that they are jointly responsible for looking after your information and using it properly. Both organisations will keep identifiable information about you for at least 15 years after the study has finished.

Your rights to access, change or move your information are limited, as your information needs to be managed in specific ways in order for the research to be reliable and accurate. If you withdraw from the study, Sheffield Teaching Hospitals NHS Foundation Trust and University of Leeds will keep the information about you that they have already obtained. To safeguard your rights, they will use the minimum amount of personally-identifiable information possible.

Your study doctor or nurse should be your first contact for any questions about your participation in this study. If you still have questions that they cannot answer, and which are not answered by any of these documents, you can contact the Data Protection Officer at the University of Leeds, or Sheffield Teaching Hospitals NHS Foundation Trust who will investigate the matter. You can do this using any of the details below. If you do contact them, please mention the name of this study (INVEST) and the Clinical Trials Research Unit.

- The Data Protection Officer for University of Leeds can be contacted using the following details: Email: DPO@leeds.ac.uk
- Telephone number: +44 (0)113 243 1751
- Postal address for data protection issues: University of Leeds, Room 11.72 EC Stoner Building, Leeds, LS2 9JT

The Data Protection Officer for Sheffield Teaching Hospitals NHS Trust can be contacted using the following details:

- Email: sth.infogov@nhs.net

- Postal address: Information Governance, Caldicott & SIRO Support, Sheffield Teaching Hospitals NHS Foundation Trust, 2 Claremont Place, Sheffield, S10 2TB
- Telephone number: 0114 226 5151

If you are not happy with the response to any queries or complaints, or believe your information is being used incorrectly or unlawfully, you should contact the Information Commissioner's Office:

- General website: ico.org.uk
- ICO contact webpage: ico.org.uk/global/contact-us
- Telephone number: 0303 123 1113
- Postal address: Information Commissioner's Office, Wycliffe House, Water Lane, Wilmslow, Cheshire, SK9 5AF

Delete this line, then print on Trust/Hospital headed paper

Participant ID:	Initials:
Date of Birth:	Principal Investigator:
EudraCT Number: 2021-006537-19	

INVEST

Phase I study of Intravesical immunotherapy for bladder cancer patients undergoing radical cystectomy

PARTICIPANT CONSENT FORM

Please initial each box

1.	I confirm that I have read and understand the information sheet [Version ____; dated ___/___/____] for the above study and have had the opportunity to ask questions.	
2.	I understand that my participation in this study is voluntary and that I am free to withdraw at any time without my medical care or legal rights being affected. I understand that even if I withdraw from the above study, the data and samples previously collected from me will be used in analysing the results of the study and in some cases further information about any unwanted effects of my treatment may need to be collected by the study team.	
3.	I understand that my healthcare records may be looked at by authorised individuals from the study team, CTRU, regulatory bodies or Sponsor in order to check that the study is being carried out correctly.	

4.	I understand that some of the information collected about me may be shared with authorised individuals (including members of the study Safety Review Committee, regulatory agencies and pharmaceutical company F. Hoffmann-La Roche Ltd) (possibly in other countries where the data protection laws are different to those in the UK and EU) to monitor the safety of study treatment and check that the study is being carried out correctly. It will not be possible to identify you from the information that is shared.	
5.	I agree to allow any information or results arising from this study to be used for healthcare and/or further medical research including monitoring the safety of the treatment that I will receive upon the understanding that my identity will remain anonymous wherever possible.	
6.	I agree that the samples of blood and urine taken for PK/ADA analysis may be sent to a central laboratory (possibly in another country where the data protection laws are different to those in the UK and EU). I understand that strict confidentiality will be always maintained and that my year of birth and study number will be used to identify these samples.	
7.	I agree that images such as CT scans, or Urograms taken as part of standard care can be used for the study.	
8.	I agree for follow-up data regarding my health to be collected from my healthcare records at the end of the trial.	
9.	I give permission for surplus tissue samples from my cancer that have been stored in the hospital pathology laboratory to be retrieved and used for the study.	
10.	I agree to a copy of this Consent Form being sent to the Clinical Trials Research Unit (CTRU) at the University of Leeds.	
11.	I agree that my GP, or any other doctor treating me, will be notified of my participation in this study.	
12.	I agree to take part in the study.	

The following points are OPTIONAL.

Even if you agree to take part in this study, you do not have to agree to this section.

Please <u>initial</u> in either the yes or no box

		Yes	No
13.	I give permission for extra blood, urine and tissue samples to be taken to be used in future translational research, which may include genetic testing. I understand that these samples are a 'gift' that may be used in future research that receives ethical approval.<br><br>I agree to these samples being stored and used for additional research investigations in a Human Tissue Authority (HTA) Licensed storage facility. I understand that strict confidentiality will be always maintained and that a unique study number will be used instead of my name and individual details will not be stored with my samples (i.e., they will be anonymised).<br><br>I agree to these samples and data collected from them being shared on a collaborative basis with researchers in the UK and potentially outside the UK, including commercial organisations.		
14.	I agree to have two MRI-PET scans, one prior to study treatment, and one following study treatment. I understand that these will have any details that could identify me removed before they are sent to be analysed. These scans will be securely provided to University of Sheffield, who will carry out this analysis.		

Participant:

Signature…………………………………………………………………………………

Name (block capitals) ……………………………………………….…………………

Date……………………………………………….…………………………………

Investigator:

I have explained the study to the above-named participant, and they have indicated their willingness to participate.

Signature…………………………………..………………………………………

Name (block capitals) …………………………………………….…………………

Date……………………………………………….…………………………………

(If used)Translator:

Signature…………………………………..………………………………………

Name (block capitals) …………………………………………….…………………

Date……………………………………………….…………………………………

(1 copy for participant; 1 for the CTRU; 1 held in patient notes, original stored in Investigator Site File)
